# Osteology of *Huabeisaurus allocotus* (Sauropoda: Titanosauriformes) from the Upper Cretaceous of China

**DOI:** 10.1371/journal.pone.0069375

**Published:** 2013-08-02

**Authors:** Michael D. D'Emic, Philip D. Mannion, Paul Upchurch, Roger B. J. Benson, Qiqing Pang, Cheng Zhengwu

**Affiliations:** 1 Anatomical Sciences Department, Stony Brook University, Health Sciences Center, Stony Brook, New York, United States of America; 2 Department of Earth Science and Engineering, Imperial College London, South Kensington Campus, London, United Kingdom; 3 Department of Earth Sciences, University College London, London, United Kingdom; 4 Department of Earth Sciences, University of Oxford, Oxford, United Kingdom; 5 Shijiazhuang University of Economics, Shijiazhuang, People's Republic of China; 6 Institute of Geology, Chinese Academy of Geological Sciences, Beijing, People's Republic of China; Raymond M. Alf Museum of Paleontology, United States of America

## Abstract

**Background:**

The Late Cretaceous titanosauriform sauropod *Huabeisaurus allocotus* Pang and Cheng is known from teeth and much of the postcranial skeleton. Its completeness makes it an important taxon for integrating and interpreting anatomical observations from more fragmentary Cretaceous East Asian sauropods and for understanding titanosauriform evolution in general.

**Methodology/Principal Findings:**

We present a detailed redescription of *Huabeisaurus allocotus* and a suite of anatomical comparisons with other titanosauriforms that demonstrate its validity via autapomorphies (e.g., division of some presacral vertebral laminae, reduced development of caudal ribs, the development of fossae relative to one another in caudal vertebral neural arches, high tibia-to-femur ratio). *Huabeisaurus* shares many features with other Cretaceous East Asian sauropods (e.g., pendant cervical ribs, anterior-middle caudal vertebrae with a nearly flat anterior centrum face and a concave posterior centrum face) that are absent in sauropods from other landmasses and strata, suggesting a close relationship among many of these forms within the clade Somphospondyli.

**Conclusions/Significance:**

Restudy of *Huabeisaurus* provides further evidence for the existence of a clade of somphospondylans – Euhelopodidae – mainly found in the Cretaceous of East Asia. Euhelopodidae represents a fourth example of the evolution of narrow crowns within Sauropoda, along with diplodocoids, brachiosaurids, and advanced titanosaurs (lithostrotians). Despite being known from fewer species than Diplodocoidea, Brachiosauridae, or Lithostrotia, euhelopodids possessed a broader range of tooth shapes than any of these clades, suggesting that euhelopodids exemplified a comparably broad range of feeding strategies and perhaps diets.

## Introduction

The sauropod *Huabeisaurus allocotus* was excavated from Upper Cretaceous sediments of northeast China in the 1990 s. The holotype of *Huabeisaurus* is a partially articulated individual composed of teeth, representative elements from all regions of the axial column (cervical, dorsal, sacral, and caudal vertebrae), ribs, complete pectoral and pelvic girdles, and nearly complete limbs. Due to its relative completeness, *Huabeisaurus* represents an important taxon for understanding sauropod evolution in Asia.

The original description of the species noted strong similarities between the osteology of *Huabeisaurus* and other Cretaceous East Asian sauropods, and in general, previous studies have pointed to some East Asian Cretaceous sauropod (e.g., *Nemegtosaurus*, *Phuwiangosaurus*) as the sister taxon of *Huabeisaurus.* In the 13 years since the original description of *Huabeisaurus*, 17 new sauropod species have been erected from the Cretaceous of East Asia (see lists in [1], [2]). Many authors have noted similarities among Cretaceous East Asian sauropods, often suggesting that several of these taxa belong to a clade grounded on a genus with well-known anatomy (e.g., Nemegtosauridae, [3]–[5]; Opisthocoelicaudinae, [6]; Euhelopodidae, [2], [7]; see [8] for further discussion). Cladistic support was recently presented for a Euhelopodidae that consisted of exclusively Cretaceous-aged members [2], in contrast with traditional studies and early cladistic analyses that posited the existence of a Euhelopodidae with Jurassic forms (see [5], [7] for detailed discussion). Both earlier and later analyses suggest some degree of endemism in East Asia, though its temporal duration remains uncertain. On a broader scale, an interesting evolutionary pattern has been recognized wherein all pre-Jurassic Cretaceous Asian sauropods lie outside of Neosauropoda, whereas all Cretaceous Asian sauropods are titanosauriforms [5]. Refining and explaining these paleobiogeographic patterns through time rests on detailed comparisons and comprehensive phylogenetic studies including East Asian sauropods, which are currently lacking.

With the aim of presenting comparative osteological data for *Huabeisaurus*, we examined the hypodigm firsthand at the Shijiazhuang University Museum. Below, we redescribe the anatomy of *Huabeisaurus*, highlighting similarities and differences with other Cretaceous East Asian sauropods. Based on synapomorphies recovered by existing cladistic datasets, we place *Huabeisaurus* in the wider context of sauropod evolution by exploring its lower level affinities. Finally, we compare the disparity of tooth shapes among derived sauropod clades, noting the exceptional range of shapes found within Euhelopodidae.

## Results

### Systematic paleontology

Dinosauria Owen 1842 [9]

Sauropoda Marsh 1878 [10]

Titanosauriformes Salgado, Coria, and Calvo 1997 [11]

Somphospondyli Wilson and Sereno 1998 [12]

? Euhelopodidae Romer 1956 [13] sensu D'Emic 2012 [2]

*Huabeisaurus* Pang and Cheng 2000 [14]

*Huabeisaurus allocotus* Pang and Cheng 2000 [14]

### Holotype

HBV-20001, a single, partially articulated individual comprising two teeth, four cervical vertebrae, six partial dorsal vertebrae, a sacrum composed of six vertebrae, 30 caudal vertebrae, four dorsal ribs, 13 chevrons, left and right scapulae, left and right coracoids, left radius, right ilium, left and right pubes, left and right ischia, left and right femora, left and right tibiae, left and right fibulae, held in the collections of Shijiazhuang University Museum, Shijiazhuang, People's Republic of China.

### Emended diagnosis

Autapomorphic features of *H. allocotus* (newly recognized herein except for the last feature) include: (1) posterior cervical vertebrae with divided prezygodiapophyseal lamina (i.e., PRDL-F present), (2) anterior dorsal vertebrae with divided anterior spinodiapophyseal lamina (i.e., anterior SPDL-F present), (3) postzygapophyseal spinodiapophyseal fossa (POSDF) larger than postzygapophyseal centrodiapophyseal fossa (POCDF) on anterior-middle caudal vertebrae, (4) caudal vertebrae with small caudal ribs (‘transverse processes’) that disappear around caudal vertebra eight, (5) ventral one-third of anterior-middle caudal vertebral centra expanded posteriorly, (6) two longitudinal ridges on the lateral faces of mid-caudal vertebral centra (ventral to the ridge at the neurocentral junction), (7) coracoid with tubercle near anterodorsal edge of lateral face, (8) distal end of radius about twice as broad transversely as midshaft (convergently acquired in derived titanosaurs), (9) tubercle on ischial plate projects from posterior margin, (10) ratio of tibia to femur length high (0.75; [14]).

### Type locality, horizon, and age

**Figure 1:**
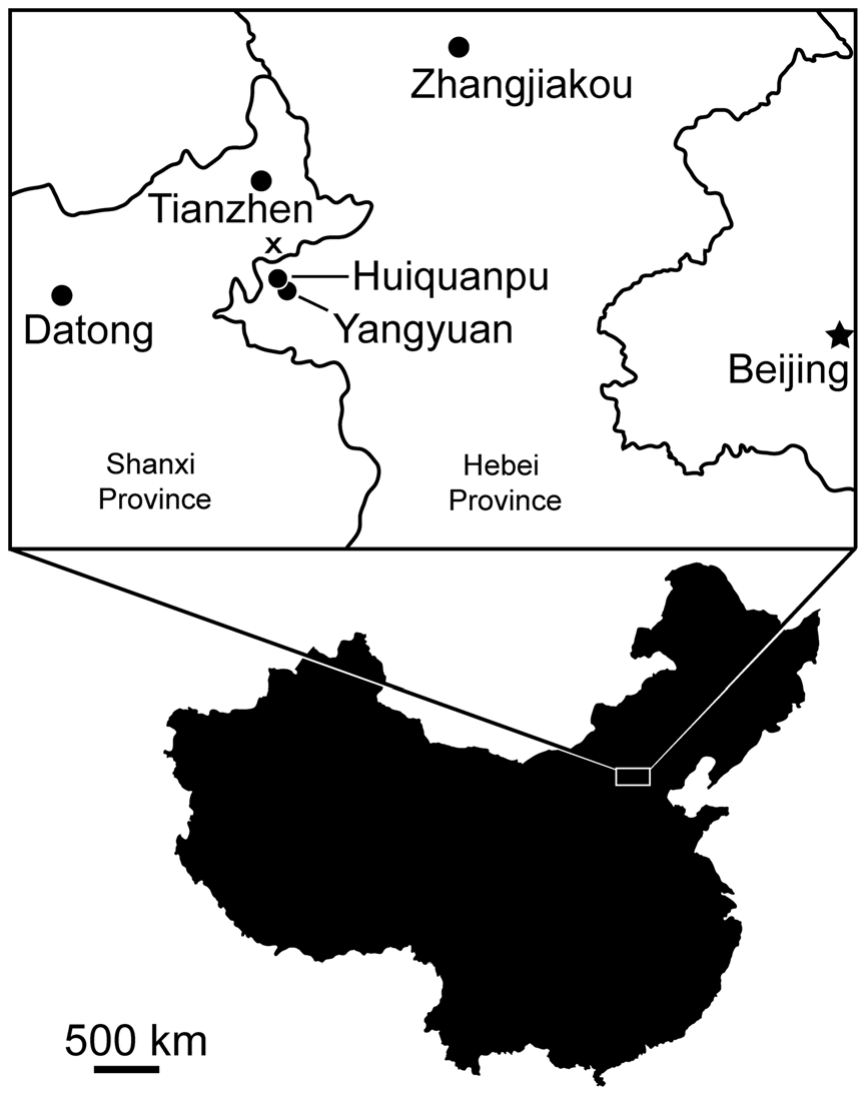
Holotype locality of *kH. allocotus* (HBV-20001) from the Upper Cretaceous of Shanxi, China, marked by ‘x’.

*Huabeisaurus* comes from Kangdailiang and Houyu, Zhaojiagou Town, Tianzhen County, Shanxi province, China (Fig. 1). The holotype was found in the unnamed upper member of the Huiquanpu Formation, which is Late Cretaceous (?Cenomanian–?Campanian) in age based on ostracods, charophytes, and fission-track dating [17], [18].

### Comments

The holotype of *H. allocotus* comprises more bones than originally listed [14] (e.g., four dorsal ribs versus one). The augmented list presented herein is based on personal observation of the skeleton in March 2012 and examination of the quarry map of the holotype located in the Museum of Shijiazhuang University. Because many of the bones of HBV-20001 are currently restored with plaster, the proposed autapomorphies above and the description that follows are based on firsthand observation confirmed by reference to pre-restoration photographs of *H. allocotus*. Our diagnosis differs from that listed in the original description [14], which used a combination of characters, nearly all of which have a broader phylogenetic distribution, to diagnose *H. allocotus*.

The isolated humerus designated the paratype [14] comes from a locality over 200 meters away from the type locality of *H. allocotus*, in a fluvially deposited sandy conglomeratic layer in the lower member of the Huiquanpu Formation (see fig. 4 in [15]). This layer is roughly 90 m lower stratigraphically than the type horizon of *H. allocotus*, which comes from the upper member of the Huiquanpu Formation. The humerus thus comes from a stratum representing a different and likely older depositional environment than that of *H. allocotus*, and does not overlap anatomically with the holotypic skeleton, and so cannot currently be referred to that taxon. The horizon that yielded the humerus also contains hadrosaurid specimens referred to cf. *Shantungosaurus* sp. and indeterminate ankylosaurid material [15].

The absence of sutures between the neural arches and centra of cervical, dorsal, and caudal vertebrae suggests that the specimen was nearing somatic maturity, but the open sutures between the scapula and coracoid and the ilium and some sacral ribs suggest that it had not reached full skeletal maturity.

### Paleoenvironment and taphonomy

**Figure 2:**
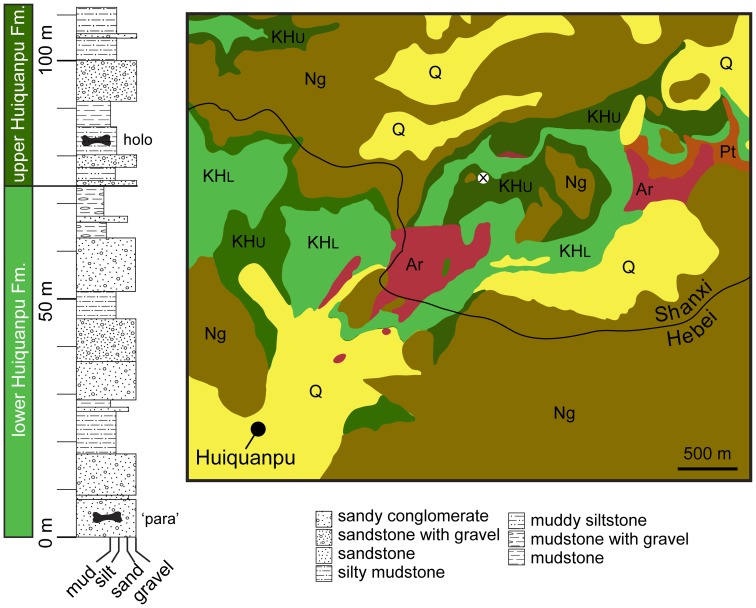
Stratigraphy and geology of holotype locality of *H. allocotus* (HBV-20001) from the Upper Cretaceous of Shanxi, China, marked by ‘x’. Based on [19]–[21]. *Abbreviations: Ar, Archean Jining Group; holo, holotype of H. allocotus; KHl, lower member of Upper Cretaceous Huiquanpu Formation; KHu, upper member of Upper Cretaceous Huiquanpu Formation; Ng, Neogene (?Miocene) Hanruoba Formation; Q, Quaternary sediments; para, former paratype humerus of H. allocotus (see text); Pt, Proterozoic rocks.*

The geology of the type locality area was described in a series of reports [14], [15], [18]–[22]. The specimen was found near the base of the upper member of the Huiquanpu Formation, in a fluvially deposited silty mudstone (Fig. 2). This locality has also produced the ankylosaur *Tianzhenosaurus youngi*, theropod material referred to cf. Szechuanosaurus campi (now regarded as a nomen dubium [23]), and indeterminate hadrosaurid material [14], [15].

**Figure 3:**
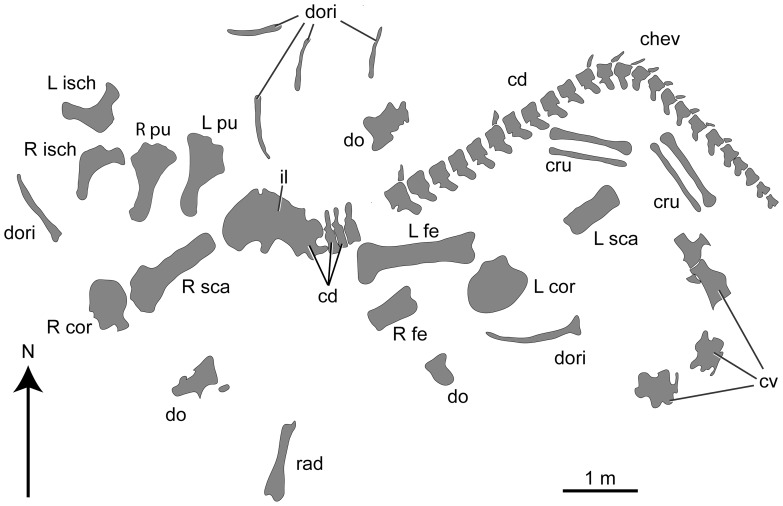
Quarry map of holotype of H. allocotus (HBV-20001) from the Upper Cretaceous of Shanxi, China. Abbreviations: cd, caudal vertebra(e); chev, chevrons; cru, crus; cv, cervical vertebrae; dori, dorsal rib(s); do, dorsal vertebra; il, ilium; L cor, left coracoid; L fe, left femur; L ish, left ischium; L pu, left pubis; L sca, left scapula; rad, radius; R cor, right coracoid; R fe, right femur; R ish, right ischium; R pu, right pubis; R sca, right scapula.

A quarry map for the holotype is depicted in Figure 3. The bones come from an area of about 10^2^ meters in a single horizon and are consistent in preservation. Limb and girdle elements from the left and right sides of the body closely match each other in size and are the appropriate size to belong to the same individual as the vertebrae and ribs. The disposition of bones in the quarry is approximately as expected if the animal were lying on its left side in an opisthotonic pose, but nearly all bones show some disorientation and disarticulation: the cervical vertebrae are arranged along a curved line, and extending along this tight curve (approximately) sit two of the dorsal vertebrae followed by the sacrum and caudal vertebrae (Fig. 3). The sacrum and first three caudal vertebrae were found in articulation and in line with the remaining articulated caudal vertebrae; others are present after a gap of about 0.5 m (Fig. 3). Twenty-seven caudal vertebrae are shown on the quarry map, but 30 were found in the collection, and pre-restoration photos indicate that 32 were originally present. Many of the chevrons were found articulated with their respective caudal vertebrae (Fig. 3). The left and right scapulae were recovered on the left and right sides of the body, respectively. The left radius was found about midway between the pectoral girdle elements. Left and right femora, pubes, and ilia were located close to one another and all of these were found near the sacrum. The sacrum is depicted with its left side facing upwards on the quarry map (Fig. 3), but this might have been an error because the left side of the sacrum is damaged and the left ilium is missing (MDD, PDM, PU, pers. obs. 2012). The elements of the left and right crura were found in close association. Dorsal rib fragments were scattered across the quarry area. In sum, some degree of transport occurred to HBV-20001 before or during burial, resulting in loss and disarticulation of some elements, but the disposition, overall agreement in size, and lack of duplication of bones suggests the presence of a single sauropod individual at this locality.

### Description

Nomenclature for vertebral laminae, fossae, and the complex sauropod sacrum follows the standardized terminology of Wilson and others [24]–[27].

### Teeth

**Figure 4:**
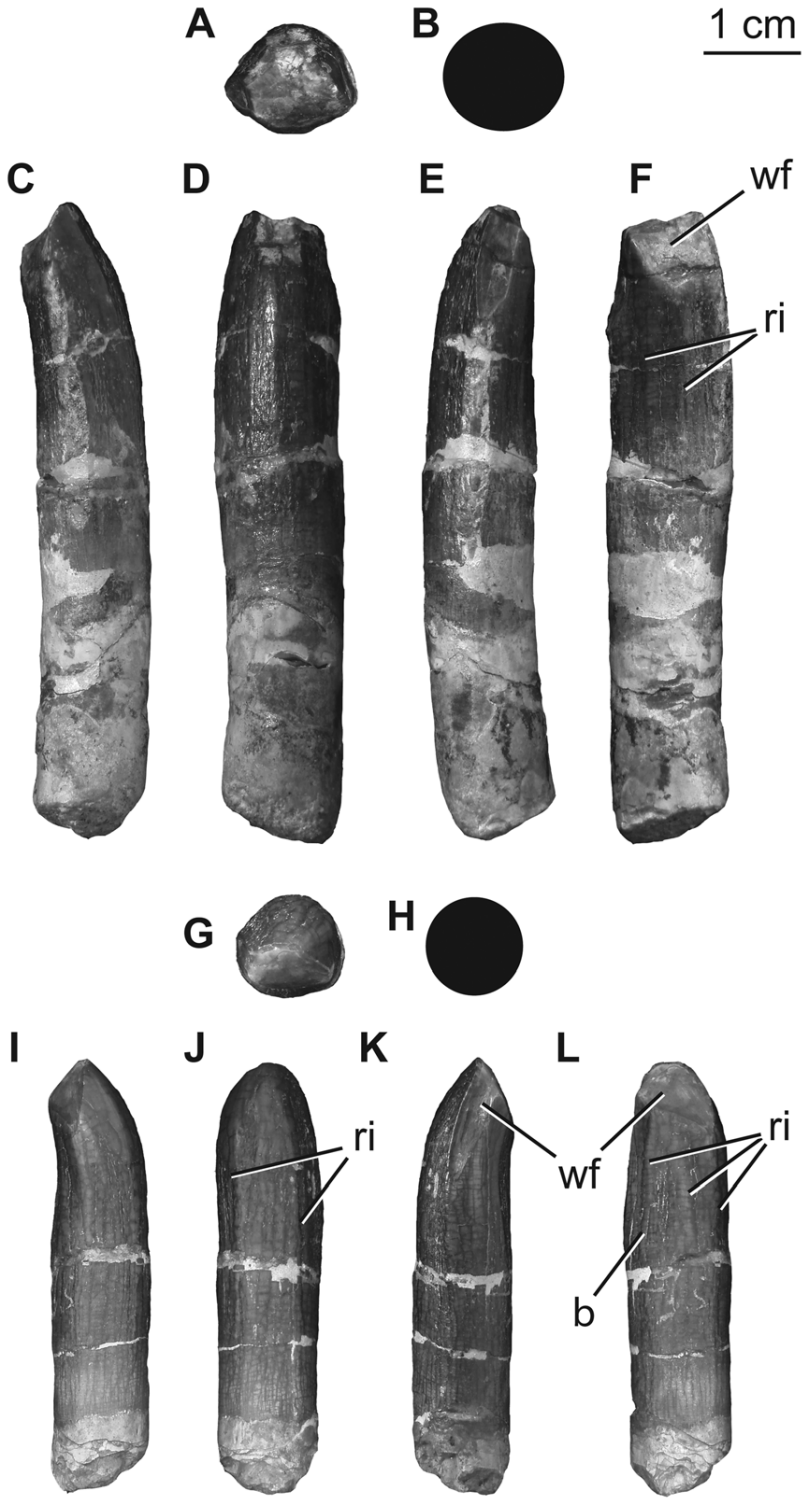
Holotypic teeth of *H. allocotus* (HBV-20001) from the Upper Cretaceous of Shanxi, China. Larger tooth in (A) occlusal, (B) basal (silhouette schematic); (C) mesial or distal, (D) labial, (E) mesial or distal, and (F) lingual views. Smaller tooth in (G) occlusal, (H) basal [silhouette schematic], (I), mesial or distal, (J) labial, (K) mesial or distal and (L) lingual views. *Abbreviations: b, bump; ri, ridge; wf, wear facet*.

One tooth was discovered in the quarry during excavation and a second was found as field jackets were opened during preparation of the specimen at Shijiazhuang University. The teeth are attributed to the holotype individual based on their close association with other bones and lack of evidence for transport from another site. Both teeth are well preserved, exhibiting wrinkled enamel. The crowns are subcylindrical (Fig. 4), with slenderness indices (maximum mesiodistal crown width divided by crown apicobasal length [28]) of 3.46 and 3.36 (Table 1), which is slightly more slender than originally described by Pang and Cheng [14] and intermediate in slenderness between broad and narrow-crowned sauropods (e.g., *Euhelopus* and *Phuwiangosaurus*). Neither tooth is strongly twisted along its length as in the upper teeth of brachiosaurids [2], [29]. Subtle, mesiodistally broad, apicobasally oriented parallel ridges extend along the lingual and labial faces of the crowns (Fig. 4). There are no labial grooves, and serrations or denticles are also absent. A very slight constriction coincides with the position of the crown-root junction; below this constriction the tooth root is subcircular in cross section. Subtle carinae extend along the mesial and distal edges of the crown. These carinae exhibit ‘prewear’, in that they do not display wrinkled enamel, but no dentine is exposed.

**Table 1:** Measurements of the teeth of *Huabeisaurus allocotus* (HBV-20001) from the Upper Cretaceous of China. All measurements are in millimeters.

Dimension	Tooth A	Tooth B
Tooth length	67.0	45.0
Crown length	45.0	37.0
Mesiodistal crown breadth	13.0	11.0
Labiolingual crown depth	11.0	9.5
Mesiodistal root breadth	12.1	10.5
Labiolingual root depth	12.0	10.0
Slenderness Index	3.46	3.36

Each tooth bears two wear facets: one oriented mesially or distally, and another oriented apicolingually (Fig. 4). The apicolingual wear facets are set at a high angle with respect to the long axis of the tooth. On the smaller tooth, a subtle bump is present at the mid-height of the crown on the lingual side, near either its mesial or distal edge (Fig. 4L).

### Cervical vertebrae

A total of four cervical vertebrae were recovered from the holotypic quarry. Two of these are fragmentary, whereas the other two vertebrae are nearly complete. According to the quarry map (Fig. 3), the two poorly preserved cervical vertebrae were articulated when found and belong to a more anterior part of the cervical series than the nearly complete vertebrae. Little anatomical information can be gleaned from the two fragmentary cervical vertebrae aside from some measurements (Table 2), so our description is based on the two better-preserved vertebrae.

**Table 2:** Measurements of cervical vertebrae of *Huabeisaurus allocotus* (HBV-20001) from the Upper Cretaceous of China.

Dimension	CvA	CvB	CvC
Centrum length (including condyle)	–	705	574
Centrum length (excluding condyle)	513	595	451
Posterior centrum height	180	209	216
Posterior centrum width	225	–	200e
Neural arch height (measured to base of postzygapophyses)	–	–	137
Neural spine height (measured from base of postzygapophyses)	–	–	265
Anteroposterior length of neural spine	–	–	188
Total vertebra height (including rib)	–	–	655

**Figure 5:**
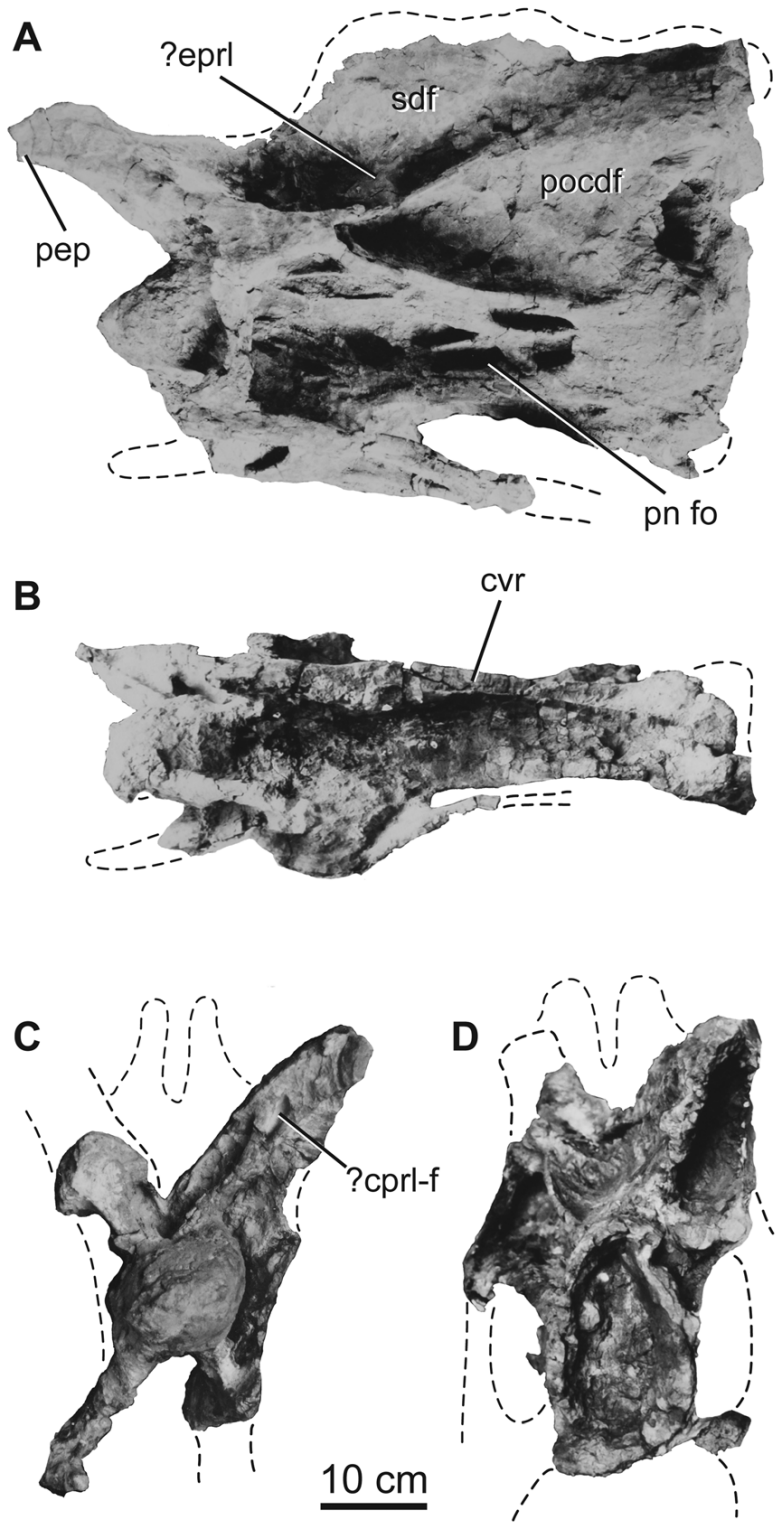
Holotypic middle cervical vertebra of *H. allocotus* (HBV-20001) from the Upper Cretaceous of Shanxi, China. Taken during original preparation in (A) left lateral, (B) ventral, (C) anterior, and (D) posterior views. *Abbreviations: ?cprl-f, possible centroprezygapophyseal lamina fossa; cvr, cervical rib; ?eprl, possible epipophyseal-prezygapophyseal lamina; pn fo, pneumatic fossa; pocdf, postzygapophyseal centrodiapophyseal fossa; pep, pre-epipophysis; sdf, spinodiapophyseal fossa.* Dashed lines indicate broken bone margins.

**Figure 6:**
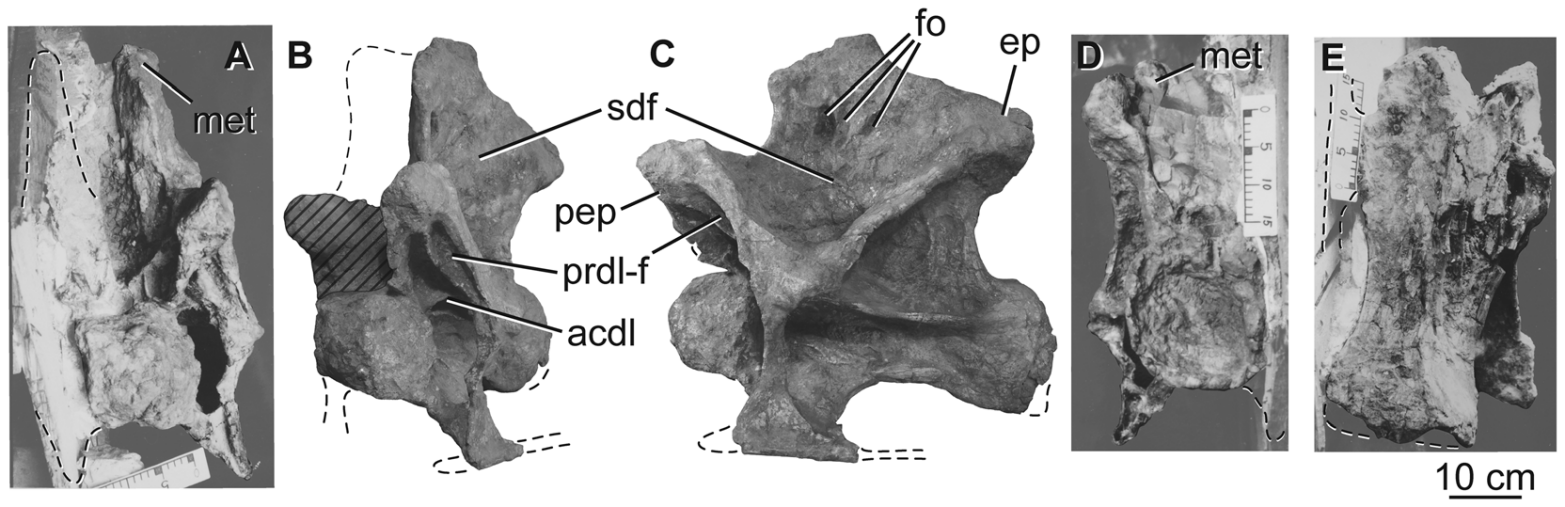
Holotypic posterior cervical vertebra of *H. allocotus* (HBV-20001) from the Upper Cretaceous of Shanxi, China. In (A) anterior, (B), anterolateral, (C) left lateral, (D), posterior, and (E) ventrolateral views. (A), (D), and (E) were taken during original preparation; (B) and (C) photographed in 2012. *Abbreviations: acdl, anterior centrodiapophyseal lamina; ep, epipophyses; fo, subfossae within spinodiapophyseal fossa; met, metapophysis; pep, pre-epipophysis; prdl-f, prezygodiapophyseal lamina fossa; sdf, spinodiapophyseal fossa.* Striped pattern indicates broken surface; dashed lines indicate broken bone margins.

All measurements are in millimeters (e  =  estimated measurement). Abbreviations: CvA, poorly preserved cervical vertebra; CvB, well-preserved middle cervical vertebra (Fig. 5); CvC, well-preserved posterior cervical vertebra (Fig. 6).

#### Middle cervical vertebra

The more anterior of the two nearly complete vertebrae (Fig. 5) belongs to the middle of the cervical vertebral series based on its elongate centrum. The vertebra is now extensively reconstructed with plaster. Pre-restoration photographs and the original description [14] suggest that it has a bifid neural spine, but we cannot definitively confirm this with firsthand observation. Damage and restoration mean that the degree of internal pneumaticity is uncertain. Most of the right side of the neural arch and spine are not preserved.

The centrum is opisthocoelous, with an aEI (average Elongation Index, sensu [29] [centrum length, excluding ball, divided by average of posterior centrum width and height]) of approximately 2.8 (Table 2). The centrum has subequal diameters at its anterior and posterior ends. The lateral face of the centrum is punctured by deep, sharp-lipped, ramifying pneumatic fossae and foramina (“pleurocoels”). The ventral surface of the centrum is concave transversely, lacks laminae, subfossae or midline ridges, and is delimited from the lateral face of the centrum by a sharp, prominent ventrolateral ridge that extends posteriorly from the parapophysis. The presence or absence of flanges projecting from these ridges as in *Euhelopus* [7] is equivocal due to damage of the ventrolateral margins of the centrum. The dorsal surfaces of the parapophyses are not excavated by large pneumatic fossae, unlike those of several other neosauropods (e.g., diplodocids, brachiosaurids, *Haplocanthosaurus*; [28], [30]). As in other East Asian mid-Cretaceous titanosauriforms preserving middle-posterior cervical vertebrae [2], the parapophyses are deflected strongly ventrolaterally in *Huabeisaurus* (Fig. 5C).

No trace of a suture at the neurocentral junction is visible. The neural arch is currently mostly covered in plaster, but pre-restoration photographs indicate the presence of several prominent laminae (posterior centrodiapophyseal [PCDL], prezygodiapophyseal [PRDL], postzygodiapophyseal [PODL], spinoprezygapophyseal [SPRL], and spinopostzygapophyseal [SPOL] laminae) and an irregularly subdivided spinodiapophyseal fossa (SDF). Multiple, subtle anterior centrodiapophyseal laminae (ACDL) occur under the web of bone comprising the PRDL and diapophysis, subdividing the prezygapophyseal centrodiapophyseal fossa (PRCDF), similar to the condition in some other sauropods (e.g., *Sauroposeidon*, [31]; *Qiaowanlong*, [32]). Pre- and postzygapophyses and metapophyses appear to have been broken or worn before recovery based on pre-restoration photographs; and they are only visible in some views in these photographs (Fig. 5). Despite this breakage, it is apparent that prezygapophyses were elongate, extending far beyond the anterior margin of the condylar ball, whereas postzygapophyses extended to about the posterior margin of the centrum. The medial margins of the prezygapophyseal processes give rise to ventromedially directed intraprezygapophyseal laminae (TPRL) that meet each other on the midline at the top of the neural canal opening. Together with the vertical centroprezygapophyseal lamina (CPRL), the TPRL and neural canal define large subtriangular centroprezygapophyseal fossae (CPRF) on the anterior face of the neural arch. In pre-restoration photographs, the CPRL appear to be invaded by a fossa (CPRL-Fig. 5C) near the prezygapophysis, but we cannot confirm this with firsthand observation.

The SPRL, SPOL, PRDL, PODL, and the dorsal surfaces of the diapophyses delimit the large spinodiapophyseal fossa (SDF). There are several deeper coels and fossae in the SDF, especially in the lower part, immediately above the diapophysis and PRDL. The presence of epipophyses, an epipophyseal prezygapophyseal lamina (EPRL), or median tubercle between the metapophyses in this vertebra are all uncertain because of damage. Pre-restoration photos (Fig. 5) suggest that there was a single thick, subvertically oriented lamina as in some other East Asian sauropods (which may represent an incipient EPRL; e.g., *Qiaowanlong, Erketu* [2], [26]), but we cannot confirm this with firsthand observation.

#### Posterior cervical vertebra

The second of the well-preserved vertebrae belongs to the posterior part of the cervical series, and at least two vertebrae are missing between it and the middle cervical vertebra described above. This posterior cervical vertebra is better preserved and less restored, missing only part of the condyle of the centrum according to pre-restoration photographs (Fig. 6). As in the middle cervical vertebra, the posterior cervical vertebra is somewhat elongate and has pendant parapophyses. The neural spine is definitively bifid in this vertebra.

The centrum has an aEI of 2.4 (Table 2). The centrum is similar in anatomical details to that described above apart from the presence of a subtle midline ridge on the anterior half of the ventral surface. Sharp-lipped lateral pneumatic fossae and foramina (“pleurocoels”) are shallower than in the middle cervical vertebra and are restricted to the anterior half of the centrum. No trace of a neurocentral junction suture is visible.

The neural arch and spine are proportionally taller than in the middle cervical vertebra, representing about twice the centrum height. The prezygapophyses have flat articular faces that face dorsomedially and only extend a short distance beyond the anterior condylar ball. The CPRL descends to the centrum and is single throughout its length. The lateral part of the underside of the prezygapophysis gives rise to a well-developed, bifurcated PRDL (Fig. 6), which is an autapomorphy of *Huabeisaurus* (though a similar structure occurs in the anterior dorsal vertebrae of the basal eusauropod *Mamenchisaurus* [33]). A pre-epipophysis (following the nomenclature of Wilson and Upchurch [7]) lies at the junction of the CPRL and divided PRDL. Although Ksepka and Norell [34] followed Wilson and Upchurch [7] in describing the pre-epipophysis as originating from different laminae in *Euhelopus* and *Erketu*, the condition is the same in those taxa as in *Huabeisaurus*. The bifurcated PRDL creates a deep fossa (PRDL-F) on the anterior face of the pendant diapophysis. The diapophysis is also connected to the centrum and neural arch by ACDL, PCDL and PODL. The short, posterodorsally oriented ACDL is single, unlike the divided ACDL of the middle cervical vertebra. The region where the diapophysis meets the top of the tuberculum is marked by a small, rounded process on the exposed left side of the vertebra. Below the anterodorsally oriented, sheet-like PCDL, a sharp ridge extends from the posterior end of the PCDL to the ventral end of the ACDL near the boundary between the centrum and neural arch. The postzygapophyses have shallowly concave articular surfaces that face mainly ventrally. They are connected by an intraprezygapophyseal lamina (TPOL) that extends ventromedially to the dorsolateral margins of the posterior neural canal opening (Fig. 6). A tall, robust epipophysis caps the SPOL near each postzygapophysis (Fig. 6), similar to the morphology seen in *Erketu* and *Mongolosaurus* ([35], [36]).

The neural spine is deeply divided, with the floor of the notch lying level with the zygapophyses. The presence of a median tubercle is equivocal due to damage. The metapophyses have a straight, inclined dorsal profile in lateral view. Their cross sections are apostrophe-shaped in dorsal view, thickening posteriorly around the SPOL. In lateral view, the stout SPRL is strongly concave, whereas the SPOL is more broadly curved. No distinct epipophyseal prezygapophyseal lamina (EPRL) is present, but the lateral face of each metapophysis bears an irregularly subdivided spinodiapophyseal fossa (SDF). In particular, there are at least three small subfossae within the upper part of this fossa.

### Cervical ribs

Only the capitula, tubercula, and part of the anterior and posterior processes of the cervical ribs are preserved. The ribs are fused to their respective vertebrae (Figs 5–6). The cervical ribs are pendant, extending ventrally for a distance subequal to the height of the centrum, as in several other East Asian Cretaceous sauropods [2]. In both cervical vertebrae, the tuberculum is notably slender anteroposteriorly, especially in comparison with the capitulum. The cervical ribs are currently broken, but the original description notes that at least some originally exceeded centrum length [14].

### Dorsal vertebrae

Parts of six dorsal vertebrae are preserved: one partial anterior dorsal vertebral neural arch, one partial dorsal vertebral centrum, three posterior dorsal vertebrae that are nearly complete but currently heavily reconstructed with plaster, and one that has been plastered into the sacrum. None of the dorsal vertebrae have observable neurocentral sutures.

**Figure 7:**
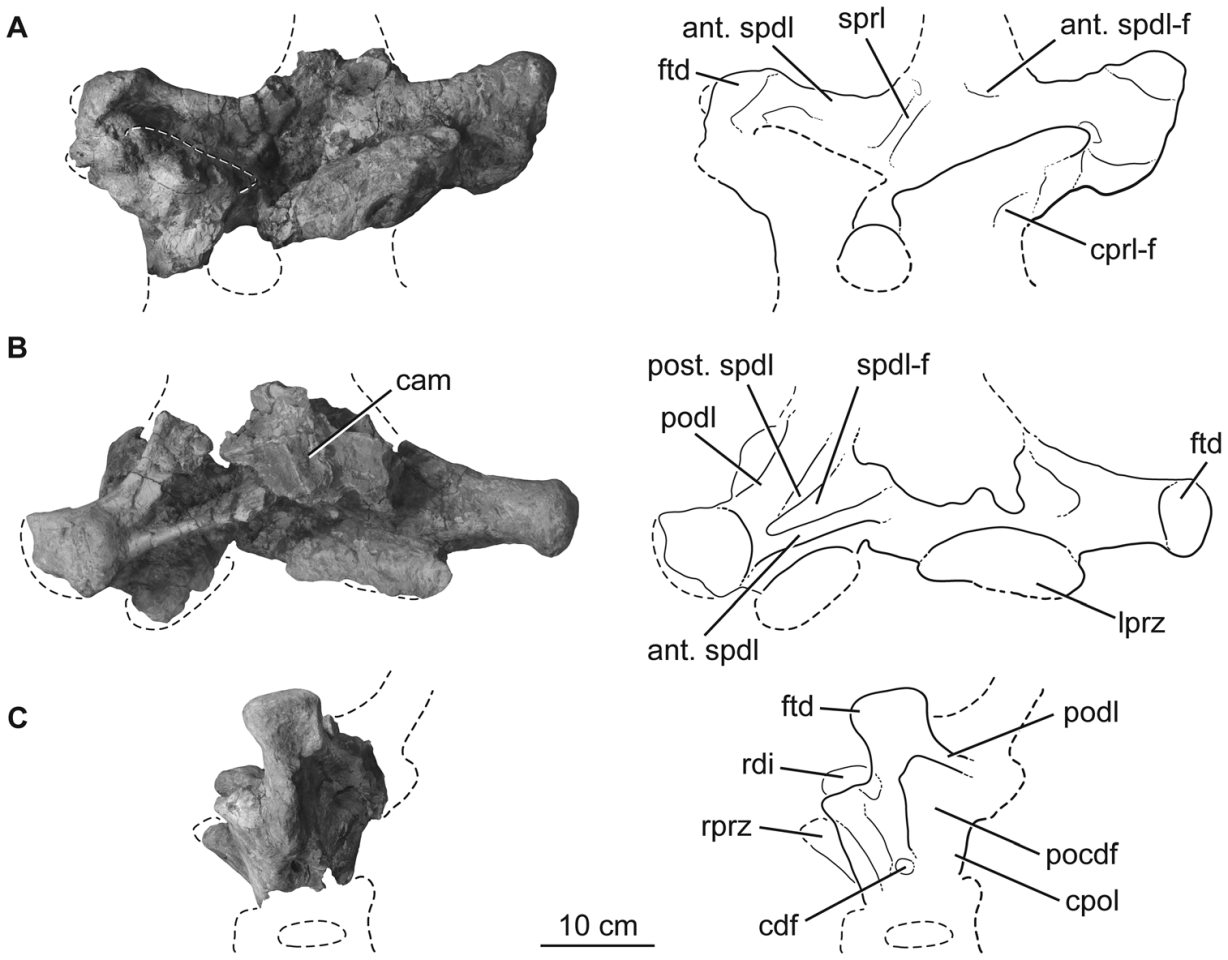
Holotypic anterior dorsal neural arch of *H. allocotus* (HBV-20001) from the Upper Cretaceous of Shanxi, China. In (A) anterior, (B) dorsal, and (C) left lateral views with interpretive line drawings. Note that the post. spdl is broken dorsally in (B). *Abbreviations: ant. spdl, anterior spinodiapophyseal lamina; cam, camellate internal pneumaticity exposed on broken surface; cdf, centrodiapophyseal fossa; cprl-f, centroprezygapophyseal lamina fossa; cpol, centropostzygapophyseal lamina; ftd, flat-topped diapophysis; lprz, left prezygapophysis; pocdf, postzygapophyseal centrodiapophyseal fossa; podl, postzygodiapophyseal lamina; post. spdl, posterior spinodiapophyseal lamina; rdi, right diapophysis; rprz, right prezygapophysis; spdl-f, spinodiapophyseal lamina fossa; sprl, spinoprezygapophyseal lamina.* Dashed lines indicate broken bone margins.

The anterior dorsal neural arch is missing its neural spine, postzygapophyses, and pedicles, and has been deformed anterolaterally (Fig. 7). The total width across the diapophyses is 483 mm. The absence of a parapophysis on the neural arch indicates that this represents one of the first three dorsal vertebrae. This identification is also supported by the size and shape of the prezygapophyses, which have articular surfaces that are wider than long, widely spaced from each other, and placed near the front of the neural spine's base rather than on distinct peduncles. Broken surfaces reveal coarse camellate pneumaticity internally, and external laminae and fossae are well developed. Prezygapophyses are connected to the diapophyses by a thick, single PRDL. A small, shallow, elliptical CPRF sits below the prezygapophyses, bounded by wide, low-relief laminae (Fig. 7A). The lateral side of the vertebra, below the zygodiapophyseal table, bears a very small, circular centrodiapophyseal fossa (CDF) and broad, shallow, postzygapophyseal centrodiapophyseal fossa (POCDF), as in the vertebrae of the pectoral region in some titanosaurs (e.g., *Rapetosaurus* [37]). The anterior surface of the neural arch above the zygodiapophyseal table is irregularly subdivided: it lacks a prespinal lamina (at least in its preserved part) and only possesses an SPRL on the right side (Fig. 7A). The SPDL is divided into parallel anterior and posterior portions; on the right side of the vertebra these laminae are distinct from one another throughout their length, but on the left side of the vertebra these laminae are poorly developed and the posterior SPDL is united with the PODL. Between the branches of the SPDL, the SPDL-F is deep on the right side and shallow on the left side. The posterior SPDL is badly damaged on the right side. Near the midline, the anterior SPDL divides to form a small pocket-like anterior SPDL-F, which we interpret as an autapomorphy of *Huabeisaurus* (Fig. 7B). The diapophyses of this vertebra are dorsoventrally tall and ‘flat-topped’ (Fig. 7A–B), as in most somphospondylans ([2], [38]), and project mainly laterally. The distal end of the diapophysis expands to form a low, rounded, anteriorly projecting process. Below the rounded upper region the distal end forms a subcircular, shallow concavity that faces outwards.

**Figure 8:**
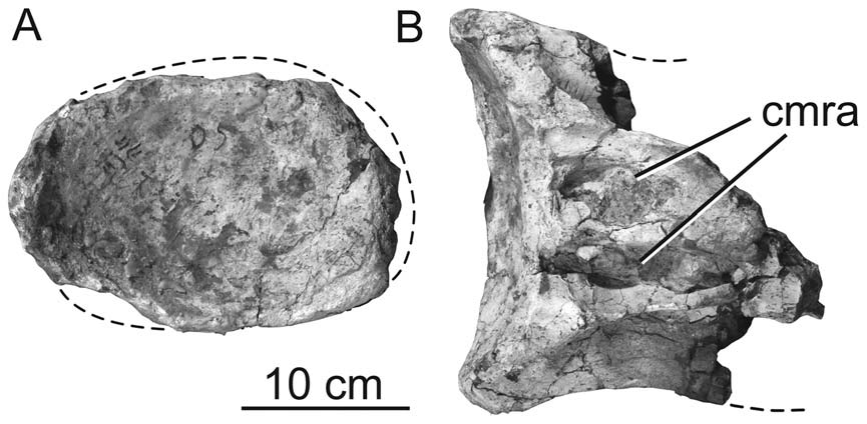
Holotypic anterior dorsal centrum of *H. allocotus* (HBV-20001) from the Upper Cretaceous of Shanxi, China. In (A) posterior and (B) dorsal views. *Abbreviations: cmra, camerate internal pneumaticity exposed on broken surface.* Dashed lines indicate broken bone margins.

The fragmentary dorsal vertebral centrum is opisthocoelous and 1.5 times wider than tall (Fig. 8). Its anterior, dorsal, and lateral faces are broken, revealing coarse camellate internal pneumaticity consisting of branching multi-centimeter scale chambers that nearly fill the centrum (Fig. 8B). It is too incomplete to determine its position in the dorsal vertebral sequence.

**Figure 9:**
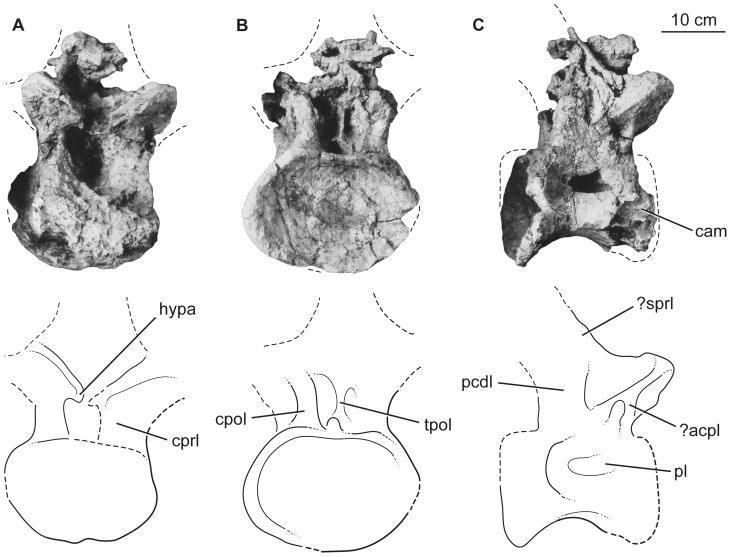
Holotypic dorsal vertebra of *H. allocotus* (HBV-20001) from the Upper Cretaceous of Shanxi, China. Photographs and line drawings of dorsal vertebra in (A) anterior, (B) posterior, and (C) right lateral views. (A–C) were taken during original preparation. *Abbreviations: ?acpl, ?anterior centroparapophyseal lamina; cam, camerate internal pneumaticity exposed on broken bone surface; cpol, centropostzygapophyseal lamina; cprl, centroprezygapophyseal lamina; hypa, accessory ventral articulation of hypantrum; pcdl, posterior centrodiapophyseal lamina; pl, pneumatic foramen (pleurocoel) in centrum; ?sprl, ?spinoprezygapophyseal lamina; tpol, ventral strut of intrapostzygapophyseal lamina.* Dashed lines indicate broken bone margins.

**Figure 10:**
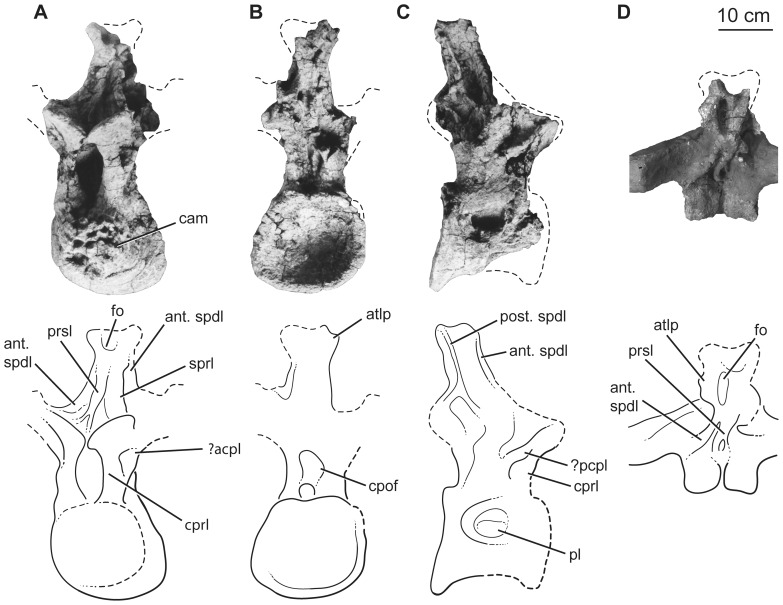
Holotypic dorsal vertebra of *H. allocotus* (HBV-20001) from the Upper Cretaceous of Shanxi, China. Photographs and line drawings of middle-posterior dorsal vertebra in (A), anterior, (B) posterior, (C), right lateral, and (D), dorsal views. *Abbreviations: ant. spdl, anterior spinodiapophyseal lamina; atlp, anterior transverse lateral process; cam, camerate internal pneumaticity exposed on broken bone surface; cpof, centropostzygapophyseal fossa; cprl, centroprezygapophyseal lamina; fo, fossa; ?pcpl, ?posterior centroparapophyseal lamina; post. spdl, posterior spinodiapophyseal lamina; sprl, spinoprezygapophyseal lamina; pl, pneumatic foramen (pleurocoel) in centrum; prsl, prespinal lamina.* Dashed lines indicate broken bone margins.

The three posterior dorsal vertebrae are more complete but are largely reconstructed with plaster. Pre-restoration photos only exist for two of these vertebrae (Figs. 9–10), so our description focuses on these. Both of these vertebrae (Figs. 9–10) are missing parts of their neural spines, transverse processes, zygapophyses, and the anterior faces of their centra. Both centra were likely opisthocoelous based on their deeply concave posterior cotyles. The more anterior of the two vertebrae (Fig. 9A–C) is 1.4 times wider than tall with a centrum length nearly equal to its width, whereas the more posterior vertebra has centrum diameters that are subequal (Table 3). The ventral surfaces of the centra are broad and gently convex transversely, without developing a ventral keel. The centra have camellate pneumaticity; the state of pneumaticity in the neural arches is unknown. The lateral pneumatic foramen (pleurocoel) is undivided, slightly acuminate anteriorly and posteriorly, and not set in a fossa in the centrum of the more anterior dorsal vertebra (Fig. 9C). In contrast, the foramen in the posterior dorsal vertebra is divided by a complex set of laminae internally, oval (i.e., not posteriorly acuminate), and set in a fossa (Fig. 10C).

**Table 3:** Measurements of dorsal vertebrae of *Huabeisaurus allocotus* (HBV-20001) from the Upper Cretaceous of China.

Dimension	DvA	DvB
Centrum length (excluding condyle)	215	–
Posterior centrum height	203	229
Posterior centrum width	286	260
Mediolateral width of left prezygapophysis	123	83
Mediolateral width across prezygapophyses	276	178

All measurements are in millimeters. Abbreviations: DvA, dorsal vertebra in Figure 9; DvB, dorsal vertebra in Figure 10.

Parapophyses and the laminae that connect them to other landmarks are poorly preserved, covered with plaster, and/or difficult to recognize in pre-restoration photographs in both vertebrae, precluding definitive identification of laminae and fossae delimited by them. On the lateral faces of the vertebrae, the PCDL do not bifurcate towards their ventral ends, unlike the condition in many somphospondylans [2], [11].

The large prezygapophyses of both vertebrae have flat articular surfaces that face dorsally and slightly medially at approximately 25° to the horizontal. Each prezygapophysis curves ventrally near the midline, indicating the presence of a hypantrum. The region surrounding the hyposphene is damaged or covered in all three vertebrae (Figs. 9–10), but it was probably present, as is the case in most sauropods excluding derived titanosaurs and many rebbachisaurids [11], [33], [39], [40]. On both sides of each vertebra, a stout CPRL extends dorsally and curls medially to support the medial part of the prezygapophysis from below. Between these CPRL there is a slot-like excavation (left and right CPRF) above the anterior neural canal opening. The stout centropostzygapophyseal laminae (CPOL) bound the CPOF medially; these fossae are separated on the midline by a well-developed ventral strut of the TPOL.

**Figure 11:**
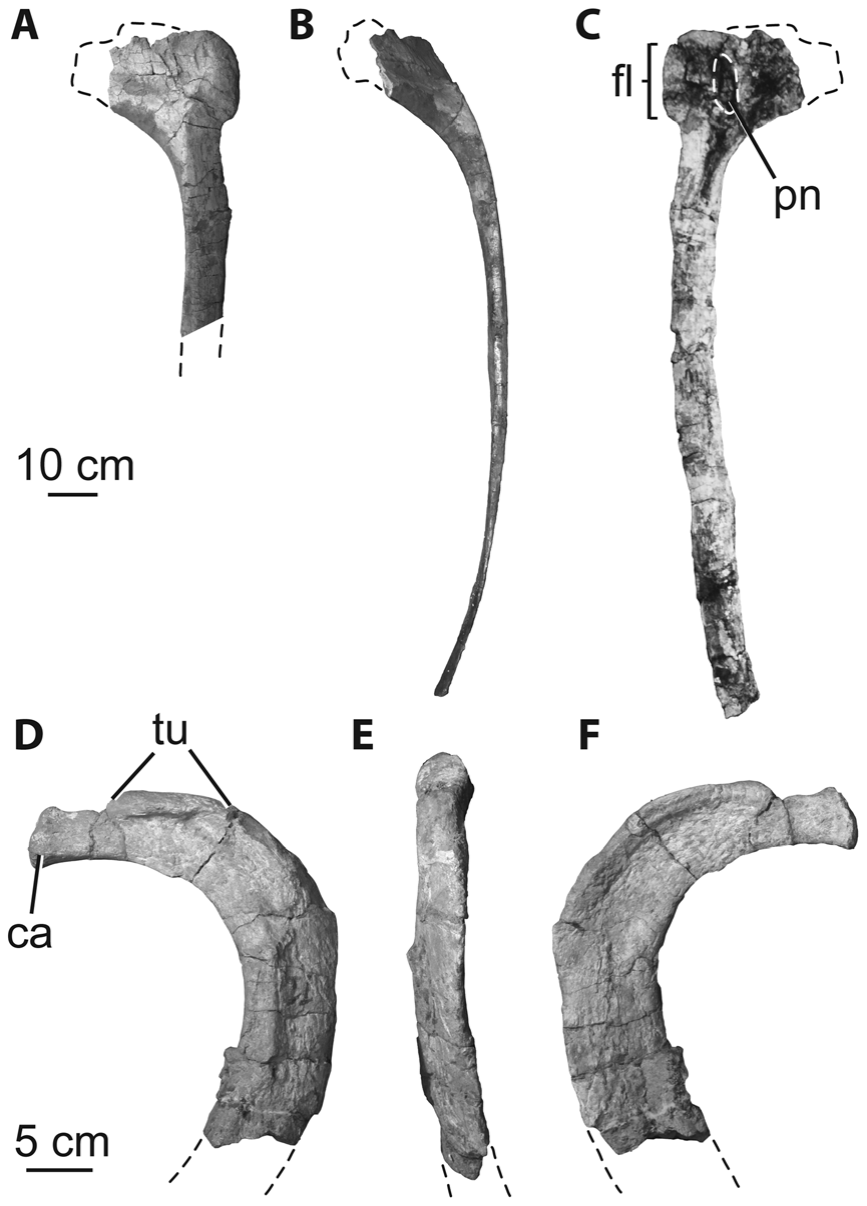
Holotypic dorsal ribs of *H. allocotus* (HBV-20001) from the Upper Cretaceous of Shanxi, China. Anterior dorsal rib in (A) lateral, (B) posterior; and (C) medial views. Posterior dorsal rib in (D) lateral, (E) posterior, and (F) medial views. (C) was taken during original preparation, all others were taken in 2012. *Abbreviations: ca, capitulum; fl, flange; pn, pneumatic opening; tu, tuberculum.* Dashed lines indicate broken bone margins.

Laminae and fossae of the neural spine are only readily identifiable on the more posterior dorsal vertebra (Fig. 10). Anteriorly, a single prespinal lamina (PRSL) extends for almost the entire length of the neural spine, except near its base, where it bifurcates slightly. Laterally the SPRF are bounded by anterior SPDL that terminate in the space between the prezygapophyses and diapophysis, suggesting that this is perhaps a stranded lamina in transition according to the pattern of “lamina capture” [25]. The anterior SPDL extends dorsally to an anterior triangular lateral process (Fig. 10B, D); the posterior triangular lateral process is broken. Some other sauropods appear to have separate anterior and posterior triangular lateral processes (e.g., *Phuwiangosaurus*, Wilson [25]: fig. 11E). Medial to these triangular lateral processes at the apex of the neural spine is a longitudinal fossa. Posterior to the anterior SPDL is a broad fossa (SPDL-F), bounded posteriorly by the posterior SPDL as in several other titanosauriforms [25]. The posterior face of the neural spine is damaged, but appears to have a broad POSL.

### Dorsal ribs

Six dorsal ribs are indicated on the quarry map (Fig. 3), but only one nearly complete element and three fragments could be located in the collections of Shijiazhuang University (Fig. 11). The shafts of all dorsal ribs are ‘plank-like’ (i.e. three to four times wider than broad in cross section), as in other titanosauriforms [33]. Near the articulation facets for the vertebra, the most complete dorsal rib (a left element) has a subtriangular cross section. This rib has a large, sharp-lipped pneumatic foramen proximally, as in other titanosauriforms [12], [33]. This pneumatic foramen is currently covered by a supporting jacket, but is visible through the support and in pre-restoration photographs (Fig. 11C). Opposite the capitulum, on the proximal end, the bone expands into a prominent flange as in some titanosaurs (e.g., *Pitekunsaurus*, MAU-Pv-AG-446-33; Paleontología de Vertebrados, Museo Municipal “Argentino Urquiza”, Rincón de los Sauces, Argentina MDD pers. obs. 2010). This ridge also appears to be present in some ribs of *Opisthocoelicaudia* (Borsuk-Bialynicka, 1977: plate 10, figure 4B), but we have not yet confirmed this with firsthand observation. The posterior edge of the plank-like region is very thin, whereas anteriorly the rib thickens and has a transversely rounded margin. Towards its proximal end, the shaft develops a broad, rounded ridge on its lateral face that is continuous with the anterior margin of the shaft. The best-preserved rib of *Huabeisaurus* has a curved length of 1450 mm and a straight length (i.e., a line from one extreme to the other) of 1420 mm.

Another dorsal rib (Fig. 11D–F) probably pertains to one of the last dorsal vertebrae based on its small size and the short distance between the tuberculum and capitulum. Its shaft is also plank-like in cross section. The other two fragmentary ribs are broken portions of shafts.

### Sacrum

**Figure 12:**
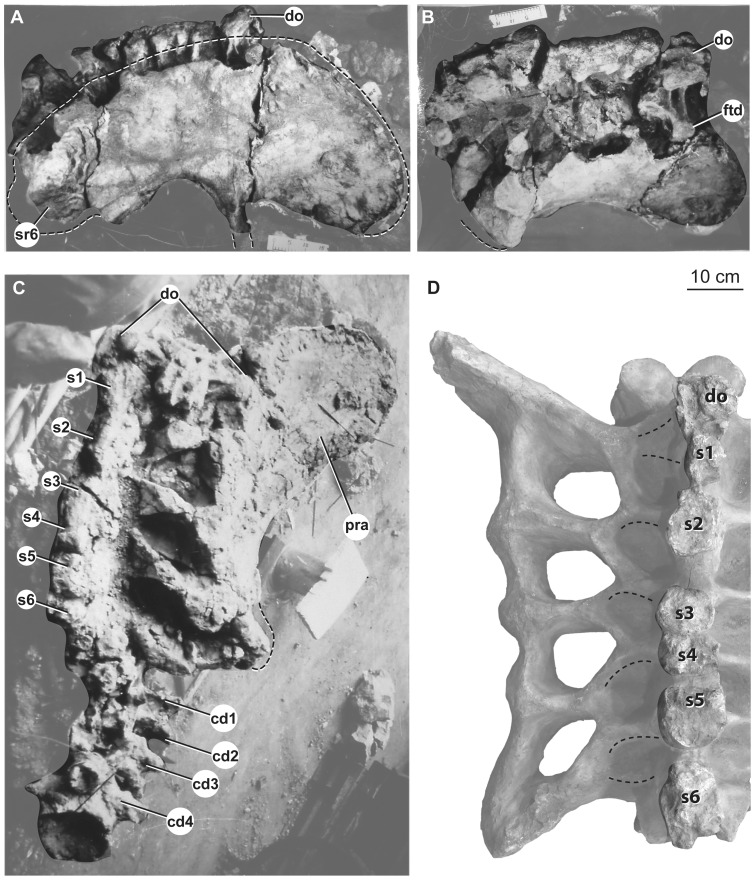
Holotypic sacrum *H. allocotus* (HBV-20001) from the Upper Cretaceous of Shanxi, China. In (A) right lateral, (B) right dorsolateral, (C) dorsal, and (D) dorsal views. (A–C) depict the sacrum during original preparation; (D) depicts the sacrum in 2012. *Abbreviations: cd1–4, caudal vertebrae 1–4; do, dorsal vertebra; ftd, flat-topped diapophysis; pra, preacetabular process; s1–6, sacral vertebral neural spines 1–6.* Dashed lines in (A–C) indicate broken bone margins; dashed lines in D indicate general locations of the last dorsal rib and the six sacral ribs.

A nearly complete sacrum consisting of six vertebrae was recovered from the quarry, originally only lacking some ribs (Fig. 12). The original description of *Huabeisaurus* suggested that only five sacral vertebrae were present based on the number of sacral ribs and intercostal foramina. The sacrum is currently heavily restored with plaster, but pre-restoration photographs show the sacrum in two oblique dorsal views and right lateral view (Fig. 12A–C). These photographs reveal that the last dorsal vertebra was taphonomically shifted posteriorly and to the right, crushing the right first sacral rib (Fig. 12A–C). Pre-restoration photographs and the number of broken sacral neural spines currently visible (Fig. 12D) indicate that the sacrum was composed of at least six vertebrae. The first vertebra crushed into the sacrum could represent a seventh sacral vertebra; because the ribs of this dorsal vertebra are not observable firsthand or in photographs, we cannot verify whether or not these ribs contacted the ilium. We interpret this vertebra as the last dorsal vertebra because: (1) its neural spine does not appear to be fused to the neural spine posterior to it, and (2) the usual sacral vertebral count for all but the most basal somphospondylans is six (with seven vertebrae in *Neuquensaurus* as the only derived exception [41], [42]).

The sacrum is only slightly longer along its centra than wide across its ribs, as in most sauropods (Table 4). The pre-restoration photographs suggest that at least the alar arms (sensu [27]) of the second through fourth sacral ribs were fused to the right ilium, and that the neural spines of these vertebrae were fused into a broad plate as in some titanosaurs (e.g., *Epachthosaurus* [43]; *Malawisaurus* [44]). The broken tops of the neural spines of the sacrum reveal that neural spines 3–5 fused in contact with one another at this level, whereas pre- and postspinal laminae separate the other sacral neural spines. Pre-restoration photographs indicate that the postacetabular process of the ilium is missing, exposing the iliac articular faces of the fifth and sixth sacral ribs (Fig. 12A–C). The corrugated texture apparent in lateral view on the fifth and sixth sacral ribs suggests a cartilaginous attachment, implying that they were not fused to the ilium at the time of death. However, we cannot confirm this based on firsthand observation.

**Table 4:** Measurements of the sacrum of *Huabeisaurus allocotus* (HBV-20001).

Dimension	Measurement
Posterior width of last sacral centrum	295
Total anteroposterior length of sacral neural spines	840
Maximum mediolateral width of sacral neural spines	114
Maximum mediolateral width of sacrum	1037

All measurements are in millimeters.

The first sacral vertebra bears a prominent convex anterior ball, whereas the last sacral vertebral centrum is flat to slightly concave posteriorly. These articular surfaces are approximately subcircular in outline. The centra of sacral vertebrae 1–6 are fused together and their boundaries are marked by low, rounded ridges. There are no obvious pneumatic fossae or foramina, but these might be filled and covered with matrix or plaster. The centra of the middle sacral vertebrae do not narrow considerably compared to the first and last sacral vertebrae, unlike the situation in some titanosaurs (e.g., *Neuquensaurus* [2], [42]). As far as can be ascertained from the restored sacrum, the ventral surfaces of sacral centra are convex transversely.

There is no evidence that a postspinal lamina (POSL) was present on the posterior midline of the neural spine of sacral vertebra 6. This spine also seems to lack the lateral laminae observed on spines 1–5.

On pre-restoration photographs, flat-topped alar processes are visible on the sacral vertebral diapophyses, as in the sacra (and dorsal vertebrae) of most somphospondylans [2], [38]. All five ribs have acetabular arms that are fused laterally to form a well-developed sacricostal yoke. Intervertebral foramina, transverse foramina, and intracostal foramina (sensu [27]), if once present, are now filled by plaster. The size and shape of the dorsal and ventral intercostal foramina have also been modified by plaster restoration. Vertebral laminae above the zygodiapophyseal table (i.e., SPRL, SPDL, SPOL) are also mostly covered by plaster.

### Caudal vertebrae

**Figure 13:**
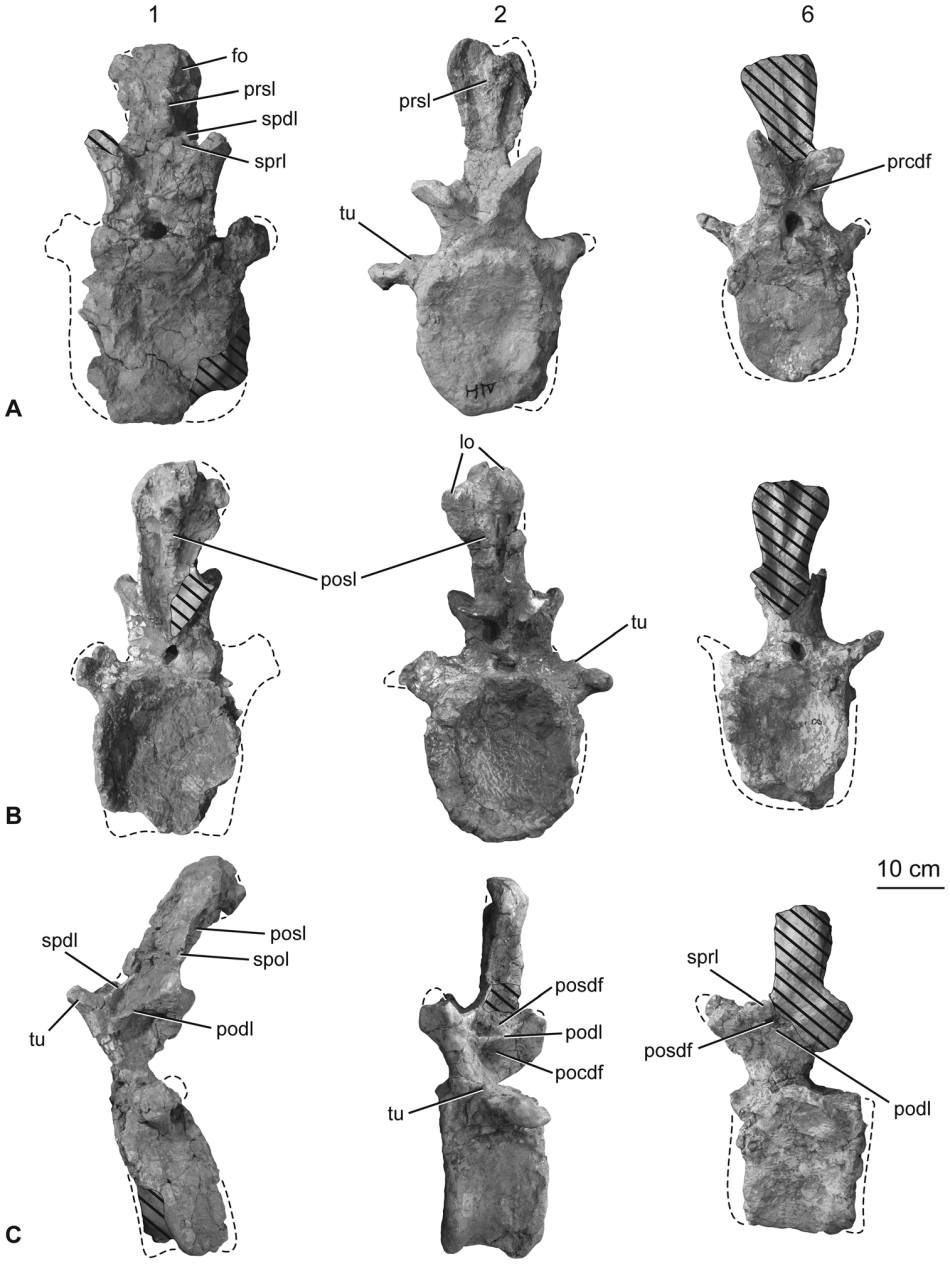
Holotypic anterior caudal vertebrae of *H. allocotus* (HBV-20001) from the Upper Cretaceous of Shanxi, China. In (A) anterior; (B) posterior, and (C) left lateral views. First, second and sixth caudal vertebrae are depicted from left to right in each row. Lateral view of second caudal vertebra reversed. *Abbreviations: di, diapophysis; fo, fossa; lo, lobe; tu, tubercle; pocdf; postzygapophyseal centrodiapophyseal fossa; podl, postzygodiapophyseal lamina; posdf, postzygapophyseal spinodiapophyseal fossa; posl, postspinal lamina; prcdf, prezygapophyseal centrodiapophyseal fossa; prsl, prespinal lamina; spol, spinopostzygapophyseal lamina; sprl, spinoprezygapophyseal lamina; tu, tubercle.* Striped pattern indicates broken surface; dashed lines indicate broken bone margins.

**Figure 14:**
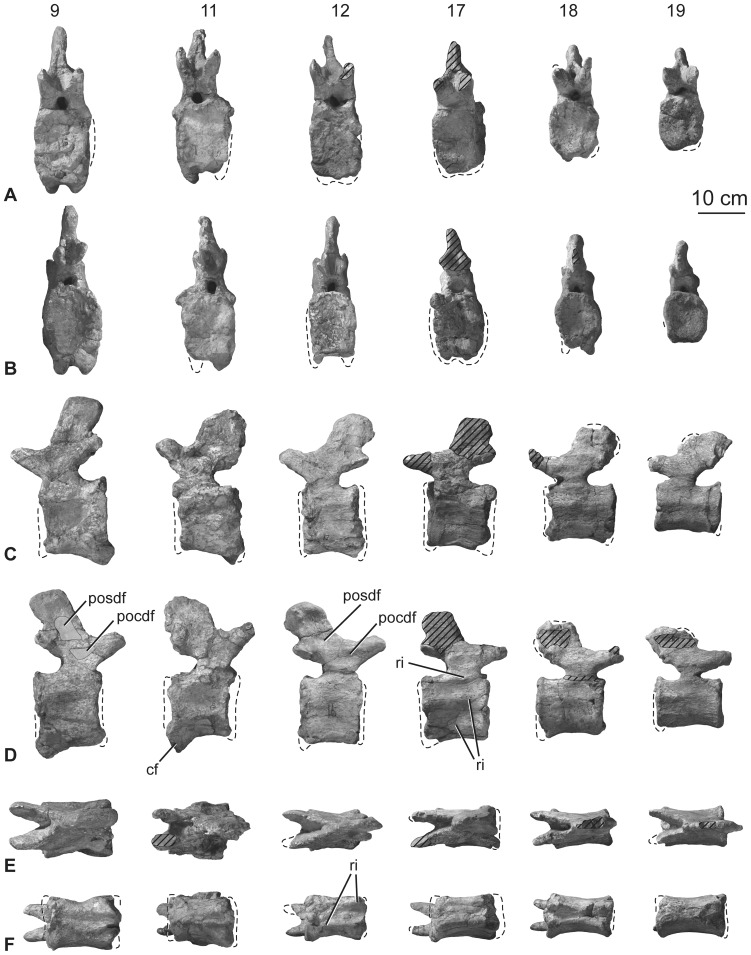
Holotypic middle caudal vertebrae of *H. allocotus* (HBV-20001) from the Upper Cretaceous of Shanxi, China. In (A) anterior; (B) posterior, (C) left lateral, (D) right lateral, (E) dorsal, and (F) ventral views. Ninth, eleventh, twelfth, seventeenth, eighteenth, and nineteenth caudal vertebrae are depicted from left to right in each row. *Abbreviations: cf, chevron facet; pocdf; postzygapophyseal centrodiapophyseal fossa; posdf, postzygapophyseal spinodiapophyseal fossa, ri, ridge.* Striped pattern indicates broken surface; dashed lines indicate broken bone margins.

**Figure 15:**
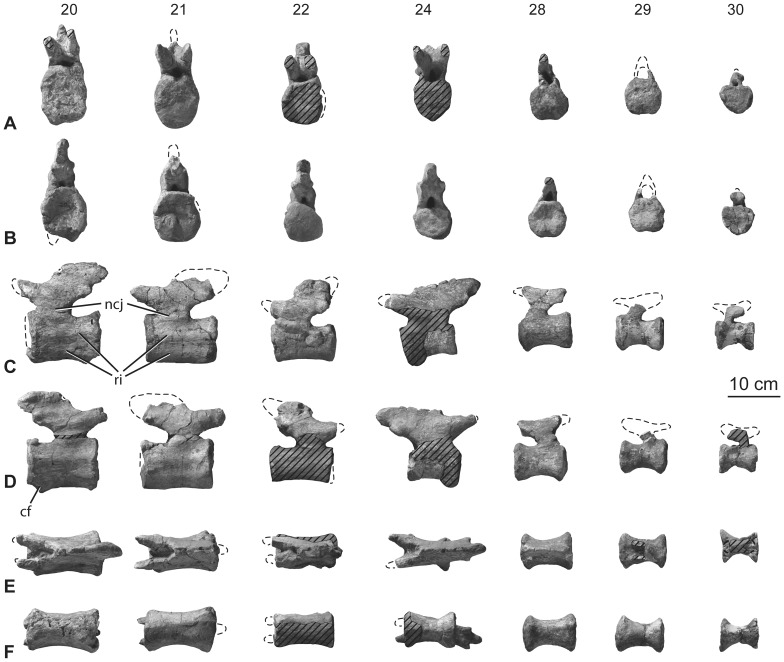
Holotypic posterior caudal vertebrae of *H. allocotus* (HBV-20001) from the Upper Cretaceous of Shanxi, China. In (A) anterior; (B) posterior, (C) left lateral, (D) right lateral, (E) dorsal, and (F) ventral views. Twentieth, twenty-first, twenty-second, twenty-fourth, twenty-eighth, twenty-ninth, and thirtieth caudal vertebrae are depicted from left to right in each row. *Abbreviations: cf, chevron facet; ncj, neurocentral junction; ri, ridge.* Striped pattern indicates broken surface; dashed lines indicate broken bone margins.

Although only 21 caudal vertebrae were listed in the holotype of *H. allocotus* [14], at least 27 are shown on the quarry map (Fig. 3) and 30 are present in the collections of the Museum of Shijiazhuang University. The caudal series is preserved as sequences of consecutive vertebrae with gaps: the first four are present, then at least two are missing, then eighteen vertebrae, then a gap of at least two, then one vertebra, then a gap of at least one, then seven vertebrae (Figs 13, 14, 15). Thus, representative elements of at least the first thirty-five caudal vertebrae are preserved in *H. allocotus*. The last preserved caudal vertebra still has a well-developed neural arch, suggesting that at least fifteen vertebrae are missing from the end of the tail based on comparisons with *Gobititan* (IVPP 12579; Institute of Vertebrate Paleontology and Paleoanthropology, Beijing, China [MDD, PDM and PU pers. obs. 2012]). Thus, the true number of caudal vertebrae in *H. allocotus* was probably close to 50, as in most sauropods (e.g. *Camarasaurus,* which has 53 [45], and *Shunosaurus*, which has 44 [46]), with the exception of the lengthened tails of diplodocids and shortened tails of some derived titanosaurs [28], [47]–[49]. Here, we refer to the 30 caudal vertebrae as ‘Cd1–Cd30’, with these numbers reflecting their sequence and position as preserved. Below, we divide the available specimens into ‘anterior’ (caudal vertebrae 1–8) and ‘middle’ (caudal vertebrae 9–30) sections based on the presence and absence of caudal ribs, respectively. We abbreviate caudal vertebra 1, 2, etc., as Cd1, Cd2, etc.

#### Anterior caudal vertebrae

The first eight caudal vertebrae are generally well preserved, although there are missing parts and some features are hidden by plaster. In particular, part of the centrum of Cd1 is heavily restored, the spine of Cd4 is damaged, the arch and spine of Cd5 are heavily restored with plaster, and the centrum of Cd8 is strongly crushed transversely.

The original description described all caudal vertebral centrum articulations as amphicoelous, with the posterior concavity slightly deeper [14]. This is true for the anterior region of the tail – anterior articular surfaces of the anterior-middle caudal vertebral centra (Cd 1–12) are shallowly concave, whereas the larger, posterior surfaces are more strongly concave (Fig. 13), a feature that has been interpreted as incipient opisthocoely ([6], but see Discussion below). Centra are taller than wide and in lateral view are short anteroposteriorly compared to their height (Table 5). For example, the length to posterior height ratio for Cd2 is about 0.5. Chevron facets are poorly preserved in the first few vertebrae, supporting the view that these are from the anteriormost region of the tail, since these are lacking in articulated sauropod specimens. In Cd1–5, the ventral surface of the centrum is broad and flat transversely and mildly concave anteroposteriorly. In Cd5 a shallow midline groove is present between the posterior chevron facets. All caudal vertebral centra are solid, lacking pneumatic fossae or foramina below or into the caudal ribs.

**Table 5:** Measurements of the caudal vertebrae of *Huabeisaurus allocotus* (HBV-20001) from the Upper Cretaceous of China.

Cd	CL	ACH	ACW	PCH	PCW	DFA	DFP	NAH	PRE	NSH	NSL	NSW	CRW
1	126	250	232	253	268	∼0	29	∼62	∼0	303	99	158	177#
2	132	270	241	254	250	22	40	87	∼0	270	76	139	350
3	154	–	–	254	270	–	–	–	–	–	–	–	–
4	159	255	237	243	241	24	55	56	23	290	75	91	225#
5	154	213	190	224	186	27	50	–	–	–	–	–	–
6	154	197	161	173	173	–	65	–	35	–	–	–	136#
7	161	172	151	167	142	–	67	100	55	–	–	–	–
8	162	–	127	171	138	–	51	68	–	–	–	–	–
9	156	141	122*	165	131	10	60	53	63	155*	–	–	–
10	147	155	119	169	122	10	67	–	51	–	–	–	–
11	150	151	128*	161	134	20	50	–	48	146	121	29	–
12	146	159	117*	138	112*	–	45	–	–	–	103	33	–
13	149	142	–	149	–	22	54	–	40	–	–	–	–
14	139	143	123	150	–	20	55	52	48	–	–	–	–
15	147	150	119	145	104	23	60	51	46	–	–	–	–
16	156	137	121	141	131	–	59	–	45	–	–	–	–
17	154	136	–	139	110	–	62	49	43*	–	–	–	–
18	161	120	112	130	103	20	65	36	43	110	111	41	–
19	158	118	101	120	106	30	66	43	24	90	135	30	–
20	158	118	97	–	104	25	63	43	26	83	132	26	–
21	160	109	96	110	101	30	58	39	34	–	–	–	–
22	165	98	93	101	98	20	56	35	–	97	∼108	39	–
23	–	–	–	–	–	–	–	–	–	68	122	38	–
24	96	73	68	59	71	14	10	29	19	57	–	39	–
28	105	60	68	66	72	10	41	20	–	–	85	25	–
29	86	61	66	58	62	22	38	–	–	–	–	–	–
30	78	59	70	59	65	19	43	18	–	–	–	–	–

Vertebrae 25–27 are excluded due to their poor preservation. Abbreviations: CL, centrum anteroposterior length; ACH, anterior surface of centrum dorsoventral height; ACW, anterior surface of centrum mediolateral width; PCH, posterior surface of centrum dorsoventral height; PCW, posterior surface of centrum mediolateral width; DFA, distance from anterior end of centrum to anterior end of neural arch; DFP, distance from posterior end of centrum to posterior end of neural arch; NAH, neural arch height (measured from dorsal surface of centrum up to the base of the postzygapophyses); PRE, extent of prezygapophyses beyond the anterior margin of the centrum; NSH, neural spine height (measured upwards from the base of the postzygapophyses); NSL, neural spine maximum anteroposterior length; NSW, neural spine maximum mediolateral width; CRW, caudal ribs mediolateral width (measured across the distal tips of the caudal ribs). A hash sign (#) denotes a CRW measurement based on only one caudal rib (measured from distal tip of caudal rib to midline of vertebra). An asterisk (*) denotes a measurement based on an incomplete element. Note that all centrum height measurements exclude chevron facets and centrum length measurements exclude the posteroventral expansion present in the anterior and middle caudal vertebrae. All measurements are in millimeters.

The neural arch occupies most of the length of the dorsal surface of the centrum in the anteriormost caudal vertebrae, but from Cd5 onwards it is displaced to cover the anterior part of the centrum, as in other titanosauriforms [11]. In lateral view, the prezygapophyses project steeply anterodorsally. The articular facets of the zygapophyses are flat; in the most anterior caudal vertebrae (Cd1–4) the facets form a 45-degree angle with the horizontal. Each prezygapophysis is supported ventrally by a TPRL and a CPRL. The lateral face of the prezygapophysis bears a tubercle (Fig. 13) that likely represents the serial homologue of the diapophysis in the dorsal vertebrae (see below; [40]). There is no dorsal process or tubercle on the prezygapophyseal portion of the SPRL (‘SPRL process’), whereas such a feature is found in some somphospondylans (e.g., *Phuwiangosaurus* [e.g., SM K11; Sirindhorn Museum Kalasin Collection, Changwat Kalasin, Thailand], *Tangvayosaurus* [TV2; Musée des Dinosaures, Savannakhet, Laos], MDD pers. obs. 2008). On the lateral face of the vertebra, a nearly horizontal lamina (PODL) separates two deep fossae: the POSDF above, and the POCDF below (Fig. 13). In most titanosauriforms, the POCDF is deeper and better developed than the POSDF (e.g., *Mendozasaurus* [50]), but in *H. allocotus* the reverse is true, representing an autapomorphy. The postzygapophyseal articular surfaces are large, flat, lack hyposphenes, and face ventrolaterally.

The neural spines are relatively low and subequal to centrum height (Table 5). The spine of Cd1 projects posterodorsally, whereas those of the remaining anterior caudal vertebrae are directed dorsally. In the most anterior caudal vertebrae (Cd1–6), the spine is wider transversely than anteroposteriorly throughout its length, and expands transversely towards its summit. This results in weakly developed posterolateral lobes in the first several caudal vertebrae. The SPRL create a moderately deep SPRF, which is partially divided by a PRSL. The PRSL expands transversely as it approaches the summit (the same is true for the POSL described below). The SPOL are single, thin plates that project prominently, extending to the posterolateral lobes atop the neural spine. These create a deep SPOF that occupies most of the posterior surface of the spine. There is a well-developed POSL on the midline of the SPOF, extending from a point level with the dorsal margins of the postzygapophyses to the spine summit. The spine summit was probably convex transversely to weakly trifid in the anterior caudal vertebrae, as in several titanosaurs (e.g. *Futalognkosaurus* [51] and *Xianshanosaurus* [52]). From Cd5 onwards, the summits of the spines are generally flat. In Cd7 and all subsequent caudal vertebrae, the spines are laterally compressed plates and lack PRSL and POSL, though SPRL and SPOL are present.

Caudal ribs occur only on Cd1–8, which is unusual for sauropods, whose caudal ribs typically disappear around Cd14–16 [30]. We regard this feature as a local autapomorphy of *Huabeisaurus* (caudal ribs also disappear by Cd10 in the derived titanosaurs *Opisthocoelicaudia* and *Alamosaurus* [33]). In *Huabeisaurus* the caudal ribs are tall at their bases but taper quickly to a blunt, subtriangular end more rapidly than in other sauropods, giving them a reduced appearance. The bases of the ribs typically occupy the dorsal part of the centrum and lower part of the neural arch as in other sauropods [33]. The precise orientation of some of the caudal ribs is difficult to determine because of distortion; nevertheless, in most specimens the ribs appear to have projected posterolaterally so that their distal tips lie level with or slightly posterior to the posterior margin of the centrum. Such backswept caudal ribs are considered to be a titanosauriform synapomorphy [1], [2]. The rib is supported ventrally by two low rounded ridges. One of these ridges extends ventrally and slightly posteriorly from the ventral margin of the rib to virtually the posterior margin of the centrum, and might represent a serial homologue of the posterior centroparapophyseal lamina (PCPL) of the dorsal vertebrae. The other ridge extends ventrally from the anterior face of the rib to the anterior margin of the centrum and is less steeply inclined, and possibly represents a serial homologue of the anterior centroparapophyseal lamina (ACPL) of the dorsal vertebrae. The fossa between these laminae is shallow and does not further invade the body of the centrum.

All caudal ribs appear to be fully fused to their respective neural arches and centra. The first several caudal vertebral ribs of *Huabeisaurus* have an anteroposteriorly elongate tubercle on their dorsal surface, which is best preserved in the second and fourth caudal vertebrae (Fig. 13). Similar elongate tubercles sit on the dorsal surfaces of the caudal ribs of several titanosauriforms (e.g., *Mendozasaurus* [IANIGLA-PV-065-5, −6; Instituto Argentino de Nivología, Glaciología y Ciencias Ambientales, Collección Paleovertebrados, Mendoza, Argentina], MDD pers. obs. 2008; *Baurutitan* [53]). Identifying such tubercles in various sauropod taxa is complicated by the lack of many complete caudal vertebral series of ontogenetically young sauropod individuals; such tubercles have several potential identities depending on the serial position of the vertebra and their precise location. These tubercles may represent muscle attachment sites and/or thickened sutures between separate ossification centers in a given vertebra. For example, in *Huabeisaurus*, the dorsal tubercles appear to sit fully on the caudal rib, presumably representing the insertion of epaxial musculature, whereas in *Baurutitan* they appear to represent a thickening of the dorsal costo-neurocentral suture (and possibly a serial homologue of the diapophysis). In *Mendozasaurus*, both the thickened costo-neurocentral suture thickening and a tubercle on the caudal rib are present (MDD pers. obs. 2008). In sauropods, the costocentral junction appears to fuse before the neurocentral and costoneural junctions in some taxa, as evidenced by ontogenetically young, partially fused vertebrae (e.g., *Maxikalisaurus* [54]) or vertebrae with only costoneural sutures visible and fused costocentral sutures (e.g., *Pitekunsaurus* [e.g., MAU-Pv-AG-446-25], MDD pers. obs. 2010). In other taxa, the rib fuses after the neural arch and centrum (e.g., *Bonitasaura*, MDD pers. obs. 2010).

Generally in sauropods, passing distally along the caudal vertebral sequence as the caudal ribs disappear, the costoneural junction merges with the position of the costocentral junction at the location of the neurocentral junction, leaving a roughened, elongate tubercle that sometimes contains a furrow (e.g., *Camarasaurus* [55]; *Tastavinsaurus* [56]; *Pitekunsaurus*, [e.g., MAU-Pv-AG-446-10, −11, –24], MDD pers. obs. 2010; *Huabeisaurus*, see below). Salgado et al. [11] considered this elongate tubercle to be a characteristic of some derived titanosaurs, but it has a wider phylogenetic distribution.

#### Middle and posterior caudal vertebrae

Preserved caudal vertebrae 9–30 are only slightly damaged or distorted. For example, the neural spine is heavily restored on Cd10; parts of the articular faces of the centrum of Cd11 are missing; the centrum of Cd13 has been transversely compressed by crushing; the spine summits of Cd18 and 21 are damaged; in Cd25 parts of the spine and the tips of the prezygapophyses are missing; in Cd28 some of the spine and arch are missing; and the prezygapophyses and most of the neural spines are absent in Cd29 and 30.

The middle caudal vertebral centra are solid, lacking any pneumatic features. Passing distally along the tail, the centra become more elongate relative to the size of their anterior and posterior articular faces (Figs. 14–15; Table 5). All centra are amphicoelous except Cd28 (where the vertebra is weakly opisthocoelous, with ventral parts of the articular surfaces that are weakly convex and the dorsal parts that are concave) and Cd29 (which has a weakly convex anterior face and flat posterior face). The ventrolateral margins of the centra of Cd9–18 form ridges that extend between anterior and posterior chevron facets to enclose a moderately deep and wide ventral fossa, as in many other somphospondylans [30], [33], [57]. This fossa is deepest posteriorly, though it occurs along the entire ventral surface, and is not divided by distinct laminae or ridges. The presence of ventrolateral ridges means that the ventral and lateral surfaces of the centra meet each other at an abrupt angle (greater than or equal to 90 degrees), which persists to Cd19–21. In the most distal of the preserved caudal vertebrae (i.e. Cd22 and more posteriorly), the centra become subcircular in transverse cross section (Fig. 15). Cd30 is unusual in possessing a sharp ridge that extends along the midline of its ventral surface, perhaps the result of crushing.

In anterior-middle caudal vertebrae, the posterior chevron facets are relatively large whereas the anterior chevron facets are less distinguished from one another. Chevron facets can be traced along the tail until at least Cd29. In some of the anterior-middle caudal vertebrae (e.g., Cd11), the ventral third of the centrum is subtly expanded posteriorly, giving the posterior margin a backswept appearance in lateral view (Fig. 14). This feature is regarded as an autapomorphy of *Huabeisaurus*. Chevron facets of the anterior-middle caudal vertebrae (especially Cd6–11) are unusually large for a sauropod, with a dorsoventral dimension subequal to that of the neural canal (Figs. 13–14). Hypertrophied chevron facets occur in some other East Asian Cretaceous sauropods, including *Phuwiangosaurus* (SM K11), *Tangvayosaurus* (TV2, MDD pers. obs. 2008), an unnamed specimen from Mongolia (IGM 100/3005; Mongolian Institute of Geology, Ulanbaatar, Mongolias [34]), and ‘*Huanghetitan’ ruyangensis* (41HIII-0001 [Henan Geological Museum, Zhengzhou, People’s Republic of China]; MDD, PDM, PU, pers. obs. 2012). These large posterior chevron facets of *Huabeisaurus* have a small fossa within them. From Cd24 onwards the anterior facets are slightly more prominent than the posterior ones.

In anterior-middle caudal vertebrae, the lateral surface of the centrum is divided by a variable number of anteroposteriorly oriented ridges. A single ridge occurs at about two-thirds of the height of the centrum in the anterior vertebrae of this series (e.g., Cd9, 11), whereas a second, ventral ridge is present in more posterior vertebrae in the series (e.g., Cd13–21). Furthermore, a third ridge represents the thickened neurocentral suture on approximately Cd12–25. The presence of a ridge situated approximately two-thirds of the way up the lateral surface can be observed in a wide array of sauropods (e.g. the basal eusauropod *Cetiosaurus* [58], the diplodocid *Apatosaurus* [59], and the basal titanosaur *Andesaurus* [1]), but the possession of two or more ridges is less common [58] and is regarded as a local autapomorphy of *Huabeisaurus* within Macronaria.

The neural arch is located on the anterior half of the centrum in most of the middle and distal caudal vertebrae, as in the anterior part of the series (Figs. 13–15), a feature that characterizes Titanosauriformes [11], [28]. Prezygapophyses are large and project anteriorly well beyond the anterior margin of the centrum. Their articular faces are flat and face mainly medially in Cd11 and 12. The postzygapophyses are flat facets located at the posteroventral corner of the spine. These facets typically face lateroventrally and taper ventrally towards the midline at the top of the posterior neural canal opening. Because of the erect orientation of the neural spines and the anterior location of the neural arch, postzygapophyses generally lie anterior to the posterior margin of the centrum, with few exceptions (e.g., Cd12).

In the more anterior of the middle caudal vertebrae (Cd9–18) there are well-defined SPRL that fade out at approximately mid-height on the spine. These SPRL create a small but deep prespinal fossa at the base of the spine that can be observed as far distally as Cd23 (Fig. 14–15). The prezygapophyses are linked to the postzygapophyses by a low rounded ridge that forms a ‘shoulder’ where the top of the neural arch meets the base of the neural spine. This subtle feature is absent from approximately Cd23 onwards. There is a well-developed postspinal fossa defined by SPOL that again fades out at approximately mid-length of the spine. Passing distally along the tail, this postspinal fossa and associated SPOL decrease in prominence until they disappear altogether around Cd17 and more distal caudal vertebrae. The neural spine itself is a laterally compressed, vertically oriented plate of bone in Cd9–18. The posterodorsal corner of the neural spine generally terminates posterior to or level with the posterior margin of the centrum in these vertebrae. More posteriorly in the series, the neural spine decreases in height and increases in length anteroposteriorly, developing a posterodorsal process that projects beyond the posterior margin of the centrum.

### Chevrons

**Figure 16:**
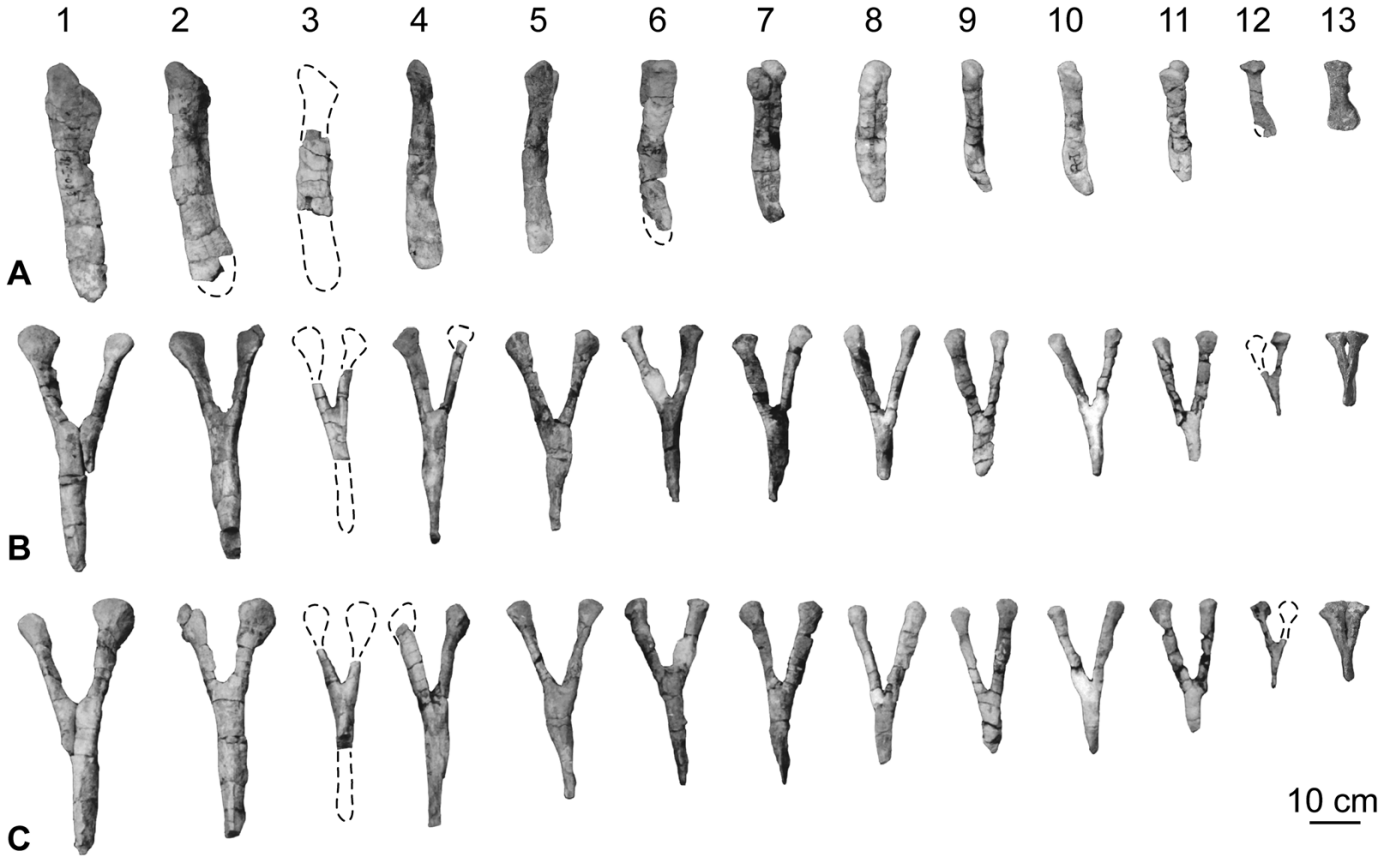
Holotypic chevrons of *H. allocotus* (HBV-20001) from the Upper Cretaceous of Shanxi, China. Photographed during original preparation in (A) lateral, (B) posterior; and (C) anterior views. Dashed lines indicate broken bone margins.

Eleven chevrons were listed in the holotype of *H. allocotus* by Pang and Cheng [14], but thirteen are visible in pre-reconstruction photographs (Fig. 16), and twelve are currently present in the Museum of Shijiazhuang University. These elements correspond to positions covering nearly the entire length of the preserved caudal vertebral series. The chevrons are generally well preserved, but there is some distortion and damage (Fig. 16). The correspondence of chevrons to particular caudal vertebrae is uncertain, so we only assign them relative numbers (Fig. 16), e.g., Ch1, Ch2 for the first and second chevrons, respectively.

The chevrons are Y-shaped in anterior or posterior view with a haemal canal that is unbridged proximally [14], although the haemal canal of the smallest preserved chevron is crushed transversely, giving it an artificially closed appearance (Fig. 16). In lateral view, the chevrons are straight or curve slightly posteriorly towards their distal ends, except in the posteriormost-preserved element, which terminates distally in a subtriangular plate formed from small anterior and posterior projections. The proximal articular surfaces are transversely compressed and anteroposteriorly ‘kinked’ in lateral view at their proximal ends, giving them contiguous but distinct anterior and posterior facets best developed in the more anterior elements (e.g., Ch5, Fig. 16). The haemal canal is proximodistally short relative to chevron length in the more anterior elements (36–44% in Chs1–4), but in the more distal elements (Chs5–12) the canal represents more than 50% of proximodistal chevron length (Table 6). The haemal canal typically represents about 30% of chevron length in most sauropods, but titanosauriforms usually have a canal length to chevron length ratio of approximately 50% [2], [33], [60]. Ventrally, the haemal canal merges into shallow grooves on the anterior and posterior faces of the blade of the chevron. These grooves grow fainter and are replaced by ridges distally on the blade. The blade of the chevron becomes transversely compressed towards its termination, but is relatively unexpanded anteroposteriorly except in the last two preserved elements in the series. A subtle but distinct projection on the posterior midline occurs along the proximal portion of the chevron blade, forming a vertically elongate ridge. As a result of this ridge, the region immediately below the haemal canal abruptly widens anteroposteriorly relative to the proximal rami. This subtle feature characterizes some other sauropod taxa (e.g., *Alamosaurus* [61]; *Phuwiangosaurus* [SM K11] and *Tangvayosaurus* [TV2; MDD pers. obs. 2008]). None of the chevrons have lateral ridges along the distal blades. There is no indication that chevrons of the middle-distal portion of the series developed the ‘forked’ or ‘skid’-like structure seen in basal eusauropods and diplodocoids [28], [30], [33]. The most distal of the preserved chevrons (Ch13) does not possess the ventral midline slit seen in ‘forked’ chevrons [28].

**Table 6:** Measurements of the chevrons of *Huabeisaurus allocotus* (HBV-20001) from the Upper Cretaceous of China.

Dimension	C1	C2	C4	C5	C6	C7	C9	C10	C12
Total dorsoventral height	368	346	317	300	253	220	246	225*	106
Maximum mediolateral width of proximal end	119	152	88	83	110	–	–	69	57
Mediolateral width of proximal ramus	41	38	33	29	25	29	22	23	29
Anteroposterior length of proximal ramus	62	62	46	43	40	43	30	37	36
Haemal canal depth	155	124	140	150	125	103	169	152	57
Haemal canal depth/total chevron height	0.42	0.36	0.44	0.5	0.49	0.47	0.69	—	0.54
Maximum anteroposterior length of distal blade	56	69	50	40	39	46	36	35	47

Numbers denote sequence as preserved, not actual sequence in vertebral series. An asterisk denotes a measurement based on an incomplete element. Chevrons 3, 8 and 11 are too incomplete to provide useful measurements. All measurements are in millimeters.

### Scapula

**Figure 17:**
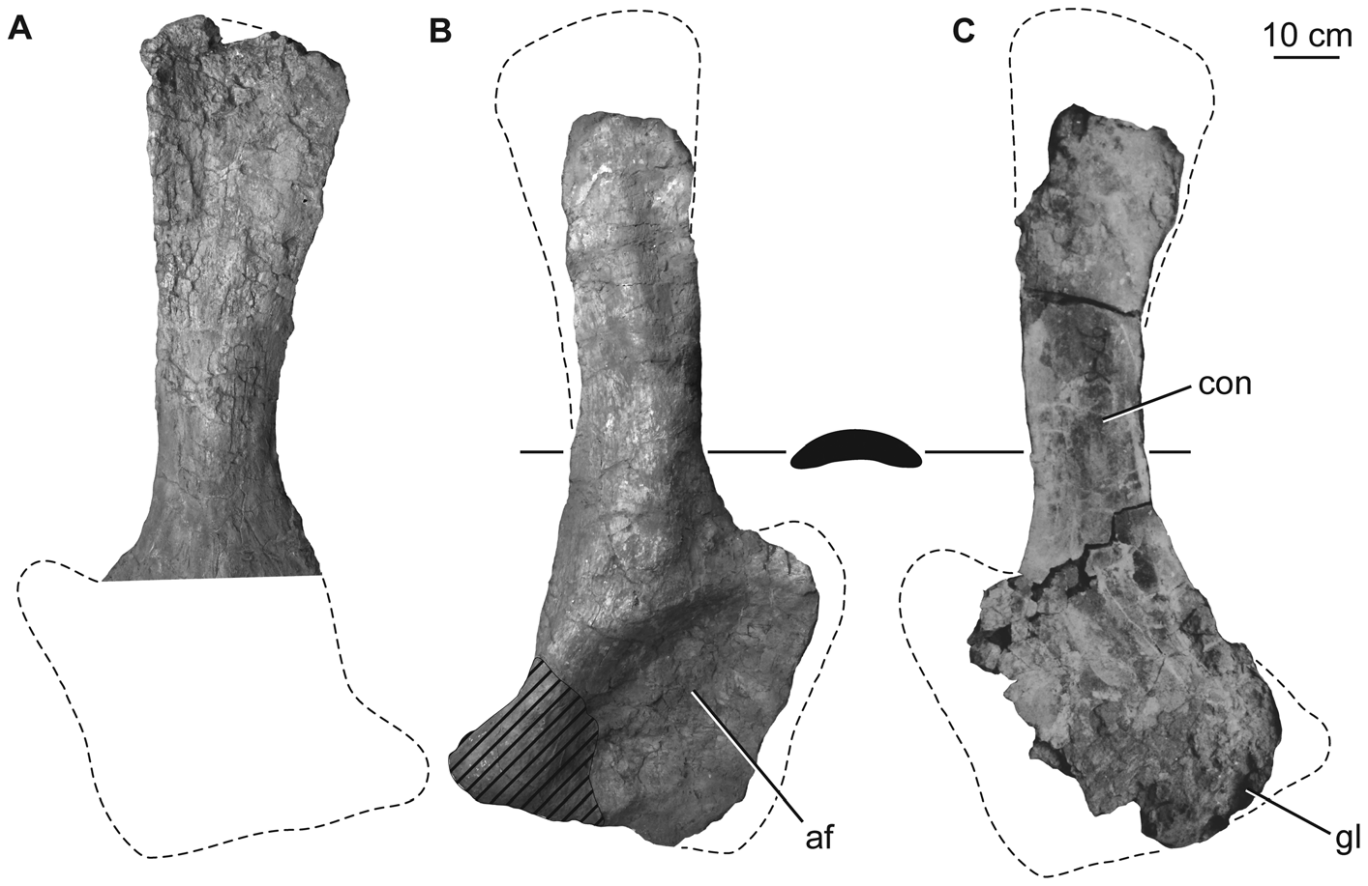
Holotypic scapulae of *H. allocotus* (HBV-20001) from the Upper Cretaceous of Shanxi, China. Left scapula in (A) lateral view; right scapula in (B) lateral and (C) medial views. Silhouette next to (B) shows schematic cross section of base of scapular blade. (A) and (B) photographed in 2012; (C) photographed during original preparation. *Abbreviations: af, acromial fossa; con, concavity, gl, glenoid.* Striped pattern indicates broken surface; dashed lines indicate broken bone margins.

Although the exact orientation of the pectoral girdle *in vivo* is uncertain, the scapulae and coracoids are generally thought to have been oblique to the major planes of orientation of the rest of the body, rendering orientational descriptors somewhat difficult to select. Here, the scapulae and coracoids will be described as if the long-axis of the scapular blade is horizontally oriented. Aside from the glenoid region, which is damaged or missing in both specimens, the preserved parts of the left and right scapulae complement one another to give a full picture of the morphology of the element. The scapulae consist of a broad proximal plate comprising an acromion and acromial fossa and a blade that forms more than half the length of the bone (Fig. 17; Table 7). The lateral surface of the acromial plate is excavated anterior to the acromial ridge and dorsal to the glenoid region. The acromial ridge is slightly posteriorly deflected, such that it is oriented at an acute angle to the long axis of the scapular blade. Immediately posterior to the glenoid articular surface, the ventral margin of the scapula is broad and convex transversely, but rapidly narrows as it merges into the base of the blade (Fig. 17). No prominent subtriangular process seems to occur along the posteroventral edge of the proximal scapula, though its absence could be due to damage. A broad ridge extends longitudinally along the proximal third of the lateral face of the blade. The dorsal margin of the blade is straight, whereas the ventral margin expands distally such that the ratio of the maximum to minimum blade dorsoventral height is 1.7 (Fig. 17, Table 7), less than the originally described value of ca. 2 [14]. The development of the acromion and distal expansion of the blade are similar to those of other somphospondylans and not as marked as in rebbachisaurids [62], [63]. The medial side of the blade is not currently observable because of the fragile nature of the scapula in the exhibit, but pre-restoration photographs (Fig. 17) and the original description [14] indicate that it was gently concave dorsoventrally. Thus, the scapular blade has a flat cross section as in most somphospondylans ([33]; Fig. 16).

**Table 7:** Measurements of the pectoral girdle and forelimb elements of *Huabeisaurus allocotus* (HBV-20001) from the Upper Cretaceous of China.

Element	Dimension	Measurement
**Right scapula**	Length	1280
	Proximal end dorsoventral height	410*
	Minimum dorsoventral height of blade	200
	Distal end of blade maximum dorsoventral height	220*
**Left scapula**	Minimum dorsoventral height of blade	200
	Distal end of blade maximum dorsoventral height	340
**Right coracoid**	Anteroposterior length	400
	Dorsoventral height	535
**Left coracoid**	Anteroposterior length	390
	Dorsoventral height	510
**Right radius**	Length	870*
	Proximal end anteroposterior length	250
	Proximal end mediolateral width	130*
	Midshaft minimum circumference	315
	Midshaft anteroposterior length	100
	Midshaft mediolateral width	95
	Distal end anteroposterior length	∼120
	Distal end mediolateral width	∼220

An asterisk denotes a measurement based on an incomplete element. All measurements are in millimeters.

### Coracoid

**Figure 18:**
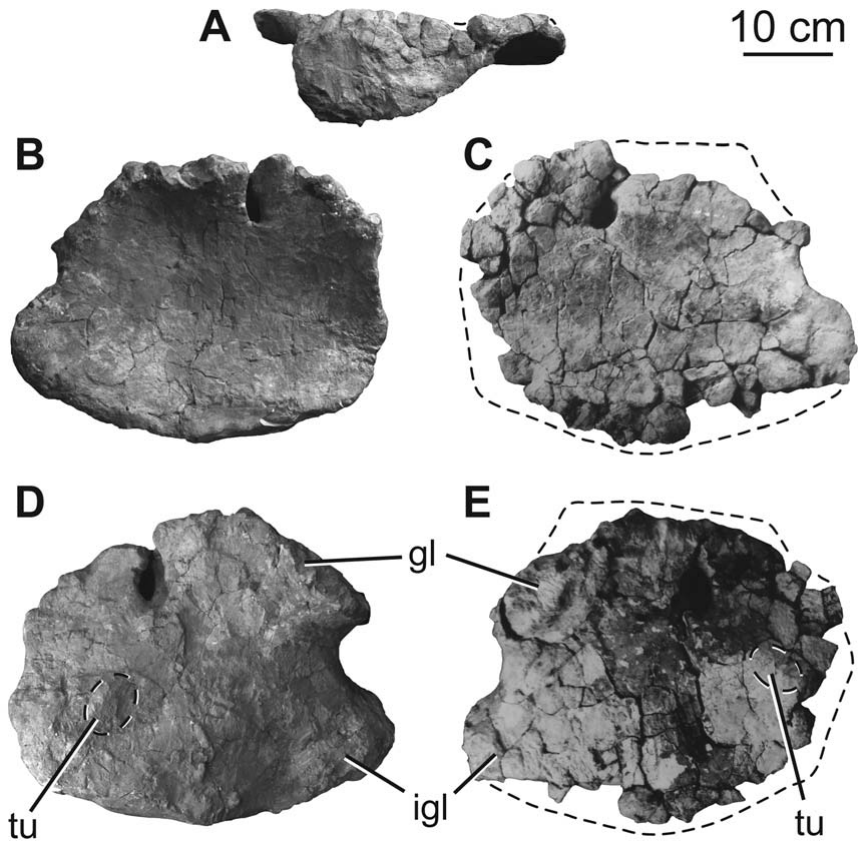
Holotypic coracoids of *H. allocotus* (HBV-20001) from the Upper Cretaceous of Shanxi, China. Left coracoid in (A) posterodorsal, (B) medial, and (D) lateral views. Right coracoid in (C) medial and (E) lateral views. (A), (B), and (D) photographed in 2012; (C) and (E) photographed during original preparation. *Abbreviations: gl, glenoid; igl, infraglenoid lip; tu, tubercle.* Dashed lines indicate broken bone margins.

Both coracoids (Fig. 18) are well preserved aside from damage around their margins (Fig. 18C, E) which has been restored with plaster (Fig. 18B, D). In articulation with the scapula, the dorsal margin of the coracoid is situated below the level of the scapular acromion plate, and the two are separated by a V-shaped notch, as is the case in all sauropods except some derived titanosaurs [28], [64]. The coracoids are dorsoventrally tall, curved plates with thick humeral and scapular articulations and well developed infraglenoid lips. Each is about 1.3 times taller than long (Table 7), as is the case for most sauropods aside from some derived titanosaurs [33]. The coracoids were not fused to their respective scapulae and each bears an essentially fully enclosed coracoid foramen. The latter is situated close to the scapular articulation, just above coracoid mid-height. It has an elliptical outline, with the long axis oriented anteroposteriorly [14]. The lateral surface of the coracoid is convex in both directions, although it becomes slightly concave anteroposteriorly towards the glenoid region. In lateral view, the anterior and dorsal margins meet at a rounded corner. This profile represents the plesiomorphic state that occurs in most sauropods, and contrasts with the derived subrectangular coracoids observed in taxa such as *Apatosaurus* and advanced titanosaurs [28], [33]. The coracoid is mediolaterally thickest at the glenoid, which has a subrectangular or nearly D-shaped outline (with the straight edge of the ‘D’ forming the medial margin) and faces posteroventrally. As in most other sauropods, the lateral part of the glenoid surface curls upwards so that it is visible in lateral view. This part of the glenoid is strongly expanded outwards so that it projects laterally well beyond the lateral surface of the rest of the coracoid. Each coracoid possesses an autapomorphic tubercle (Fig. 18D–E) on its lateral face near the anterodorsal edge of the element, just above the height of the coracoid foramen.

### Radius

**Figure 19:**
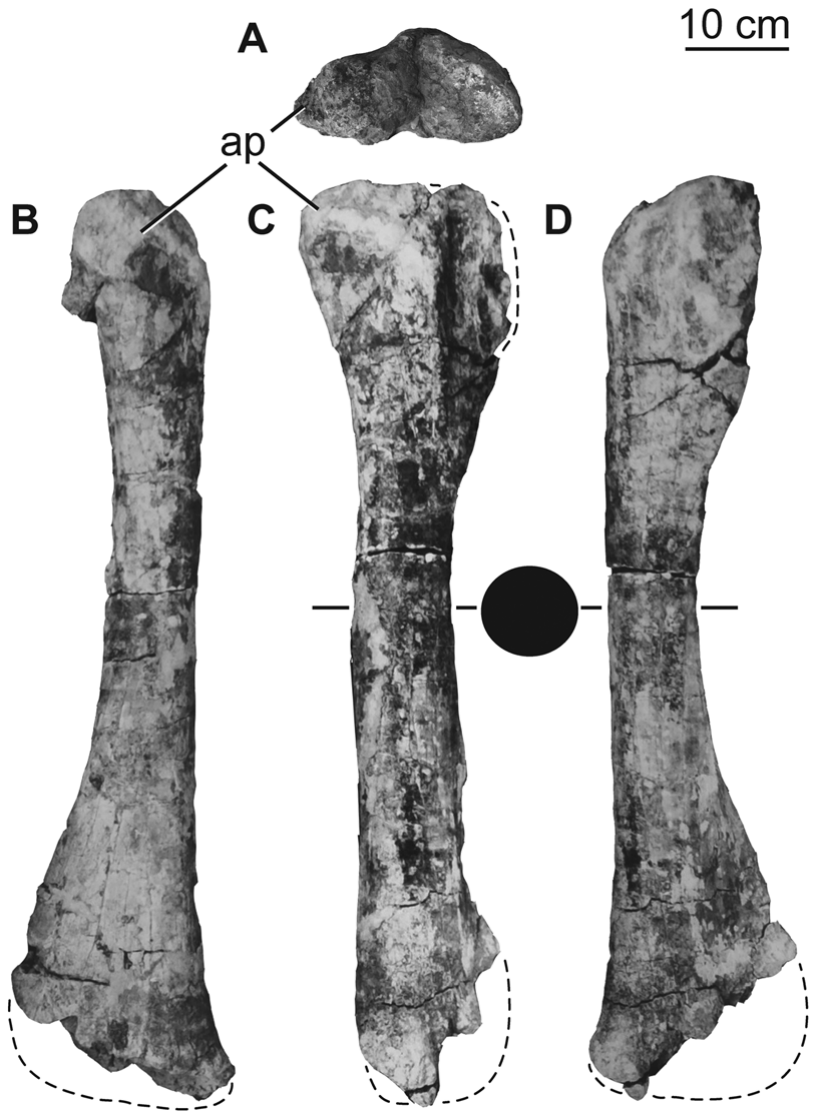
Holotypic left radius of *H. allocotus* (HBV-20001) from the Upper Cretaceous of Shanxi, China. In (A) proximal, (B) anterior, (C) lateral, and (D) posterior views. Silhouette between (C) and (D) shows schematic cross section of the diaphysis. (A) photographed in 2012; (B–D) photographed during original preparation. *Abbreviations: gl, glenoid; igl, infraglenoid lip; tu, tubercle.* Dashed lines indicate broken bone margins.

The left radius is damaged at its proximal and distal ends (Fig. 19). The radius is gracile, with a midshaft width to length ratio of 0.12 (Table 7). In anterior view, the lateral face of the shaft is straight, whereas the medial face is concave (Fig. 19). The anteroposteriorly expanded proximal end has a prominent ridge on its lateral face (Fig. 19). The proximal end has an approximately oval outline with a pointed anterior process and broadly rounded posterior process. The proximomedial margin is nearly straight, whereas the proximolateral margin is concave anteriorly and convex posteriorly. The oval cross-section of the upper shaft gradually transforms into a rounded D-shape at mid-shaft, with the long axis of the cross section extending transversely. This ‘D’ shape becomes more anteriorly compressed towards the distal end, with a transversely rounded anterior face and increasingly flattened posterior face. This is associated with the strong transverse expansion of the distal shaft and distal end of the bone, as originally described as a twisting of the bone [14]. Strong transverse expansion of the distal radius (i.e., distal end about twice as broad transversely as midshaft) is normally found only in titanosaurs (e.g., *Alamosaurus* [61], *Jainosaurus* [65]) and is considered a local autapomorphy of *Huabeisaurus*. Posterolateral ridges are weak to absent along the distal half of the bone and do not extend further proximally.

### Ilium

The right ilium has undergone substantial restoration of its margins and overall curvature. Pre-restoration photographs (Fig. 11) indicate that the ilium was elongate with a flaring preacetabular process as in other titanosauriforms [11]. The preacetabular process has since been flattened during preparation and restoration. The preacetabular process extends far anterior to the pubic peduncle. Dorsally, the ilium is incomplete, but enough of its margin is preserved to show that it possesses a preacetabular process with a low profile. The pubic peduncle is incomplete, but it is apparent that the ilium contributed about half of the margin of the acetabulum. The postacetabular process is missing (Fig. 11A–C). No evidence of pneumaticity is apparent in the ilium, but much of the surface is obscured by plaster.

### Pubis

**Figure 20:**
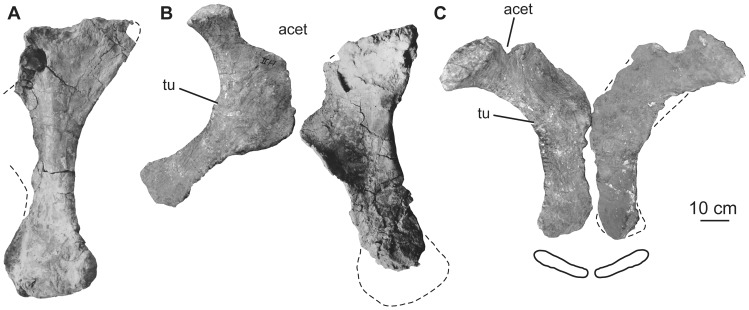
Holotypic pubes and ischia of *H. allocotus* (HBV-20001) from the Upper Cretaceous of Shanxi, China. Left pubis in (A) medial view. Right pubis and ischium in (B) lateral view (only loosely articulated to depict their size relative to one another). Articulated ischia in (C) posterodorsal view. Outlines below ischia indicate articulated view of distal ends. (A and the ischium in B) photographed in 2012; (C and the pubis in B) photographed during original preparation. *Abbreviations: acet, acetabulum; tu, tuberosity.* Dashed lines indicate broken bone margins.

Both the left and right pubes were recovered from the quarry. Parts of the pubes are currently restored with plaster, but pre-restoration photographs show that the majority of these bones are preserved (Fig. 20). Both pubes are currently displayed in field jackets; as such, their lateral surfaces cannot be observed.

The pubis is composed of an anterior plank-like body with a posterior thin, curved flange of bone that forms the floor of the pelvis. The iliac peduncle of the pubis is compressed, with its long axis about 1.7 times greater than the axis perpendicular to this (see Table 8). The iliac articular surface appears to narrow mediolaterally towards its posterior end, but it is not clear if this is a preservational artefact. Although partly reconstructed, the acetabular region slopes such that it faces dorsally and laterally, with the lateral margin forming a gently upturned rim. The proximal and anterior margins of the pubis meet at an angle of ∼60°. Despite poor preservation along this region, it is clear that no well-developed ambiens process is present. The obturator foramen enters the bone obliquely and has an elliptical outline, with the long axis oriented in the same plane as that of the pubis shaft. On the medial surface, the foramen is situated within a well-defined fossa. The articular surface for the ischium is relatively short (0.35 of pubis length; Table 8). In lateral view, the symphysis meets the ischial articulation at a sharp angle.

**Table 8:** Measurements of the pelvic girdle elements of *Huabeisaurus allocotus* (HBV-20001) from the Upper Cretaceous of China.

Element	Dimension	Measurement
**Right ilium**	Dorsoventral height anterior to pubic peduncle	570*
**Right pubis**	Length	810*
	Proximal end anteroposterior length	445
	Iliac peduncle anteroposterior length	270
	Iliac peduncle mediolateral width	160
	Dorsoventral height of ischiadic articulation	350
	Distal end anteroposterior length	340
	Distal end mediolateral width	130
**Left pubis**	Length	990
	Proximal end anteroposterior length	325*
**Right ischium**	Length	725
	Proximal end anteroposterior length	395
	Iliac peduncle anteroposterior length	205
	Iliac peduncle mediolateral width	100
	Dorsoventral height of pubic articulation	285
	Distal end anteroposterior length	208
	Distal end mediolateral width	∼48

An asterisk denotes a measurement based on an incomplete element. All measurements are in millimeters.

The medial surface of the pubis is anteroposteriorly convex along its proximal portion, but becomes anteroposteriorly concave along the middle third as a result of the medial deflection of the pubic symphysis. Distally, this medial surface appears to flatten, but neither pubis is well enough preserved in this region for this morphology to be confirmed with certainty. In medial view, the anterior margin of the pubis is concave, and a prominent anterior expansion of the distal end forms an anterior boot (Fig. 20). The left pubis preserves a flattened subtriangular area on the posteromedial surface of the distal end for articulation with the equivalent corresponding area on the left pubis.

### Ischium

Both the left and right ischia were recovered from the quarry and are essentially complete. The ischia retain their three-dimensional geometry and their morphology is unobstructed by plaster, although the left is better preserved than the right (Fig. 20). The ischium is considerably shorter than the pubis (ischium: pubis length ratio is 0.73, see Table 8, [14]). Whereas the pubes and ischia are subequal in length in most sauropods [28], [64], this shortening of the ischium occurs in most somphospondylans [2], [11], [28], [33], [66]. Aside from the robust, semicircular iliac peduncle, the ischium is formed from a thin plate of bone throughout its length. The iliac peduncle comprises about one-fifth of the proximodistal length of the element, giving the ischium a large (about one-fourth) contribution to the margin of the acetabulum. Posteriorly, the iliac peduncle narrows mediolaterally to form a triangular projection in lateral view. The iliac articular surface slopes to face dorsolaterally; it is also mildly convex anteroposteriorly (Fig. 20). The acetabular surface is wide transversely in the region of the iliac peduncle, narrows in its central region, and widens again (though not as strongly) towards the pubic articulation. This morphology has also been observed in most rebbachisaurids (e.g. *Demandasaurus*, see [67] and “*Ornithopsis eucamerotus*” from the Lower Cretaceous of the Isle of Wight, UK [68]). As in the latter taxon and the Late Cretaceous Chinese somphospondylan *Sonidosaurus* (LHV 0010; Long Hao Geologic and Paleontological Research Center, Hohhot, Nei Mongol, People’s Republic of China, PDM pers. obs. 2007), the ischium of *Huabeisaurus* possesses a prominent ridge that extends medially from the margin of the acetabulum on the section formed by the iliac peduncle.

The bone lining the posterior pelvic floor is thin and forms a deep bowl; anteriorly, this region forms the articular surface for the pubis. This articulation has a narrow subtriangular outline in anterior view, with concave dorsomedial and convex ventrolateral margins and a tapered ventromedial tip. In lateral view, the pubic articulation and the symphysis are demarcated by a change of direction: the proximal part of the symphysis extends posteroventrally towards a small transversely expanded area, which turns more steeply ventrally.

The distal shafts of the ischia are twisted so that they are nearly coplanar when in articulation (Fig. 20C). The shafts themselves approach the condition of having a straight ischial articulation, as in titanosaurs (i.e., “plate-like” ischia that lack an emargination anteroventrally (e.g., *Andesaurus* [1]; *Alamosaurus* [61]). The posterolateral face of the ischium bears a ridge-like tubercle at about mid-length as in most neosauropods [2], [28]. This tubercle differs from that of other neosauropods in that it projects out from the margin of the ischium, rather than sitting on the plate of the bone, making it visible as a kink in the profile of the dorsal margin of the distal shaft in lateral view (Fig. 20). We interpret this posterior placement and projection of this tubercle as an autapomorphy of *Huabeisaurus*. Although the distal tip of the ischium is incomplete posteriorly, the distal blade is clearly expanded transversely relative to the minimum width of the blade. The distal end is at least five times as wide transversely as thick dorsoventrally.

### Femur

**Figure 21:**
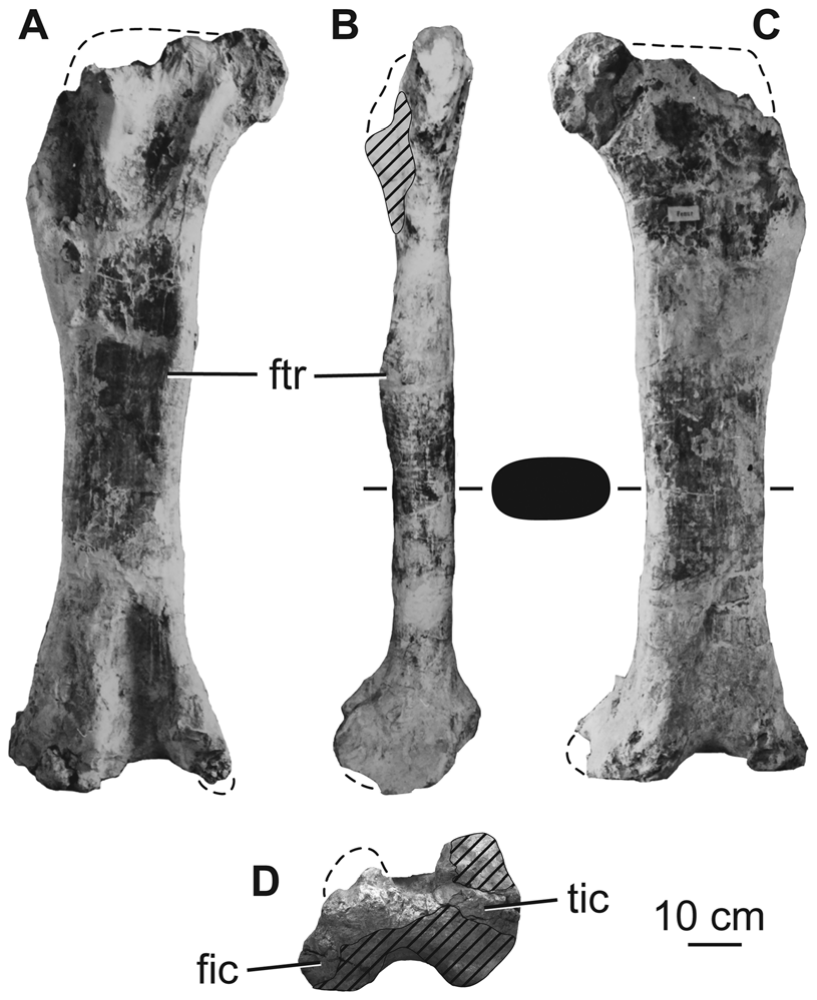
Holotypic left femur of *H. allocotus* (HBV-20001) from the Upper Cretaceous of Shanxi, China. Photographed during original preparation in (A) posterior, (B) medial, (C) anterior, and (D) distal views. Silhouette between (B) and (C) shows schematic cross section of the diaphysis. *Abbreviations: fic, fibular condyle; ftr, fourth trochanter; tic, tibial condyle.* Striped pattern indicates broken surface, dashed lines indicate broken bone margins.

A nearly complete left femur and the distal fourth of a right femur were collected from the quarry. The hindlimb was originally described as robust [14], but the femur is somewhat gracile (midshaft width divided by length  = 0.16; Table 9) and has a mediolaterally expanded, eccentric midshaft cross section (mediolateral width divided by anteroposterior breadth  = 1.7; Table 9), like most titanosauriforms [33]. The femoral head appears to project slightly dorsally, but the exact proximal outline is uncertain because of damage (Fig. 21). The proximal end bears a prominent lateral bulge, with the lateral margin above medially deflected, as in most titanosauriforms [33]. This proximolateral margin is anteroposteriorly thinner than the rest of the femur, forming a trochanteric flange towards the anterior face of the shaft. The posterior surface of the proximal end has two dorsoventrally short, nearly vertical ridges, one situated near the midline and the other near the femoral head. These define the medial edges of shallow, vertically oriented troughs, and the lateral ridge bounds the trochanteric flange. It is difficult to determine whether this posterior surface morphology is entirely genuine or the result of deformation. The fourth trochanter is situated close to the posteromedial margin of the shaft, just above the mid-length of the femur (Table 9). This trochanter is reduced to a low, rounded ridge that is not visible in anterior view. The medial surface of the fourth trochanter is gently rugose, but there is no concavity medial to this process. The anterior surface of the shaft is gently convex mediolaterally, whereas the posterior surface is flat, becoming mediolaterally convex towards its medial and lateral margins (Fig. 21).

**Table 9:** Measurements of the hindlimb elements of *Huabeisaurus allocotus* (HBV-20001) from the Upper Cretaceous of China.

Element	Dimension	Measurement
**Right femur**	Midshaft anteroposterior length	∼115
	Midshaft mediolateral width	∼265
	Distal end anteroposterior length	270
	Distal end mediolateral width	535
**Left femur**	Length	1560
	Proximal end anteroposterior length	160
	Proximal end mediolateral width	430
	Midshaft minimum circumference	645
	Midshaft anteroposterior length	145
	Midshaft mediolateral width	245
	Distal end anteroposterior length	380
	Distal end mediolateral width	465
**Right tibia**	Length	1170
	Proximal end anteroposterior length	208
	Proximal end mediolateral width	287
	Midshaft minimum circumference	430
	Distal end anteroposterior length	160
	Distal end mediolateral width	278
**Right fibula**	Length	950*
	Proximal end anteroposterior length	305*
	Proximal end mediolateral width	90
	Midshaft minimum circumference	315
	Midshaft anteroposterior length	115
	Midshaft mediolateral width	80
**Left fibula**	Length	1150
	Proximal end anteroposterior length	305*
	Proximal end mediolateral width	95
	Midshaft minimum circumference	315
	Midshaft anteroposterior length	120
	Midshaft mediolateral width	75

An asterisk denotes a measurement based on an incomplete element. All measurements are in millimeters.

The distal ends of both femora are poorly preserved but nearly complete. The fibular and tibial condyles appear to be subequal in size. There is a deep concavity on the posterior surface between the two distal condyles. The fibular condyle is poorly preserved, but clearly divided (Fig. 21). The medial face of the tibial condyle is nearly flat and faces medially and slightly proximally, as is typical for sauropods. The distal condyles are only weakly divided distally and are oriented perpendicularly with respect to the long axis of the bone, unlike the medially or laterally beveled distal femora of some titanosauriforms (see [7] for discussion). The distal articular surface is mediolaterally concave and anteroposteriorly convex, although this convexity is only strongly developed on the tibial condyle. The distal condyles do not extend onto the anterior surface of the femur. The anterior half of the distal surface, between the two distal condyles, is deflected such that it faces ventrally, but also slightly anteriorly. The anteroposterior depth of the midshaft of the femur equals slightly more than one-third of the depth of the distal condyles, as in most sauropods except derived diplodocoids [40].

### Tibia

**Figure 22:**
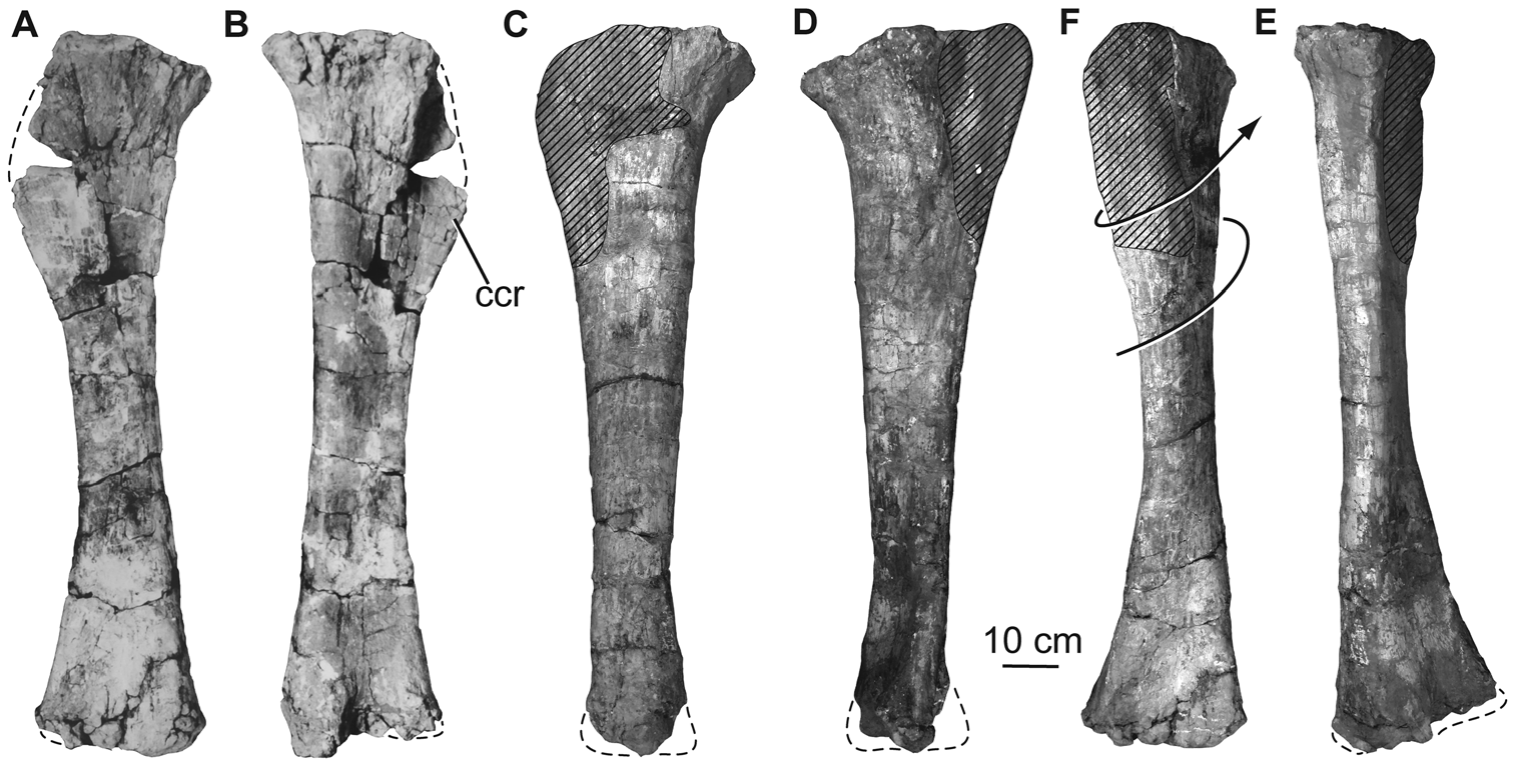
Holotypic left tibia of *H. allocotus* (HBV-20001) from the Upper Cretaceous of Shanxi, China. In (A) lateral view during original preparation, (B) medial view during preparation, (C) lateral view in 2012, (D) medial view in 2012, (F) anterior view in 2012, (G) posterior view in 2012. Twisting arrow indicates that the tibia has been incorrectly restored (views C–D) by ‘twisting’ (breaking and re-gluing/plastering) since original preparation. *Abbreviations: ccr, cnemial crest.* Striped pattern indicates broken surface, dashed lines indicate broken bone margins.

Both tibiae were recovered adjacent to their respective fibulae. The majority of their cnemial crests and the distal end of the left element are not preserved. Both tibiae have been reconstructed with an unnatural twist between their proximal and distal ends, as evident from comparing pre-restoration photographs with the tibiae on display in the exhibit (Fig. 22). The ratio of the length of the tibia to that of the femur is approximately 0.75, as noted in the original description [14]. This value is higher than in other sauropods, which typically have tibia: femur length ratios between 0.56–0.68 (see *The autapomorphic tibia: femur ratio of* Huabeisaurus in the Discussion). The tibiae are gracile elements, with a midshaft width to length ratio of 0.11 (Table 9). In dorsal view, the proximal end has a subrectangular outline, with a concave posterior margin. The proximal articular surface is weakly convex in both directions, although it forms a very gentle concavity at the anteromedial corner. The medial edge of the proximal end forms an anteroposteriorly reduced projection that overhangs the medial margin of the remainder of the tibia; this is more prominently developed in the right tibia. Based on pre-restoration photographs, the cnemial crest appears to have projected mainly laterally (Fig. 22). There is no small process in the cnemial fossa (i.e., ‘second cnemial crest’ [69]), on the lateral surface of the proximal end, posterior to the true cnemial crest).

The posterior surface of the proximal part of the tibia, approximately down to the level of the distal tip of the cnemial crest, maintains the mediolateral concavity that occurs on the posterior margin of the proximal articular end. The shaft has a flattened ‘D’-shape in horizontal cross-section, with a flat to mediolaterally concave posterolateral surface. Distally, the anterior surface of the tibia expands transversely to form the subtriangular area seen in most other sauropods. This region is mediolaterally concave, although this is almost certainly the result of crushing. Although incomplete medially, the distal end is clearly not strongly expanded transversely relative to the midshaft (Fig. 22), and is anteroposteriorly compressed, as in other titanosauriforms [11].

### Fibula

**Figure 23:**
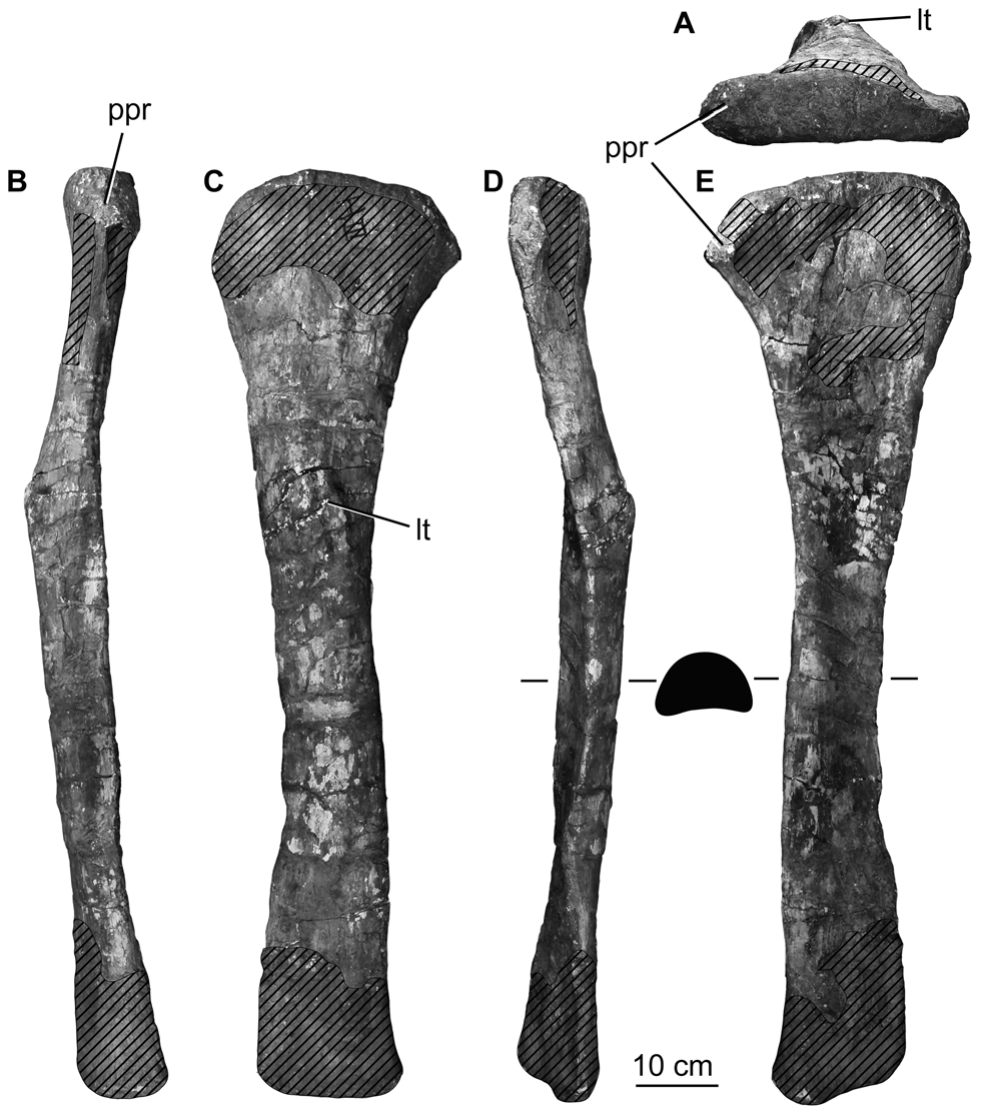
Holotypic left fibula of *H. allocotus* (HBV-20001) from the Upper Cretaceous of Shanxi, China. In (A) proximal, (B) anterior, (C) lateral, (D) posterior, and (E) medial views. Silhouette between (D) and (E) shows schematic cross section of the diaphysis. *Abbreviations: lt, lateral trochanter; ppr, posterior process.* Striped pattern indicates broken surface, dashed lines indicate broken bone margins.

Both fibulae were recovered from the quarry and are well preserved, aside from missing portions of their proximal ends (in particular the anterior margins), as well as the distal end of the right element (Fig. 23). The fibulae are gracile, with a midshaft anteroposterior breadth to length ratio of ca. 0.12 (Table 9). In medial view, the anterior margin of the fibular shaft is concave, whereas the posterior margin is straighter. The fibula is bowed laterally in anteroposterior views. In proximal view, the fibula has a weakly crescentic outline, with a slightly concave medial margin and more strongly convex lateral margin. The proximal end is mediolaterally compressed and anteroposteriorly elongate, with the posterior half much thicker mediolaterally than the anterior half. The posterior part of the proximal end expands into a subtriangular process. There is no well-developed anterior crest on the fibula as in many derived somphospondylans [7]. In lateral view, the posterior half of the proximal margin slopes ventrally, with this slope steepening along the posteriormost portion; as such, this surface faces dorsally, but also posteriorly. There is no clearly defined triangular striated area on the proximal quarter of the medial surface, but in neither fibula is this surface particularly well preserved. The medial surface is gently concave anteroposteriorly along the proximal half of the fibula, but is mainly flat along the distal half, becoming gently concave at the distal end (Fig. 23). In contrast, the lateral surface is anteroposteriorly convex throughout the length of the fibula, with this convexity most prominent along the shaft. The lateral trochanter consists of two parallel, anterodorsally oriented ridges: a smaller, anteriorly positioned ridge and a more prominent, posterior ridge, as in other neosauropods. It is situated a little above the mid-point of the shaft. The anterior margin of the distal half forms a thin ridge, whereas the posterior surface is mediolaterally thicker. There is a shallow concavity on the medial face of the distal part of the shaft just above the distal end and located anteriorly on this surface. In distal view, the fibula has a subtriangular outline, with the apex of this triangle projecting anteriorly. The lateral margin of this triangle is flat to gently convex and curves into the posterior margin, whereas the medial margin is gently concave and the posterior margin convex. The distal end is barely expanded transversely relative to the midshaft, and there is very little expansion along the distal medial margin (Fig. 23).

### Bone histology

Yao et al. [16] briefly described the vertebral, costal, and tibial bone histology of *Huabeisaurus allocotus*. The exact identity of the bones sectioned or the precise sample locations within those bones are unknown. A combination of woven, fibrous, and lamellar bone textures with occasional ‘rest lines’ and some Haversian remodeling were presented [16]. These thin sections are now lost.

### Isolated humerus

**Figure 24:**
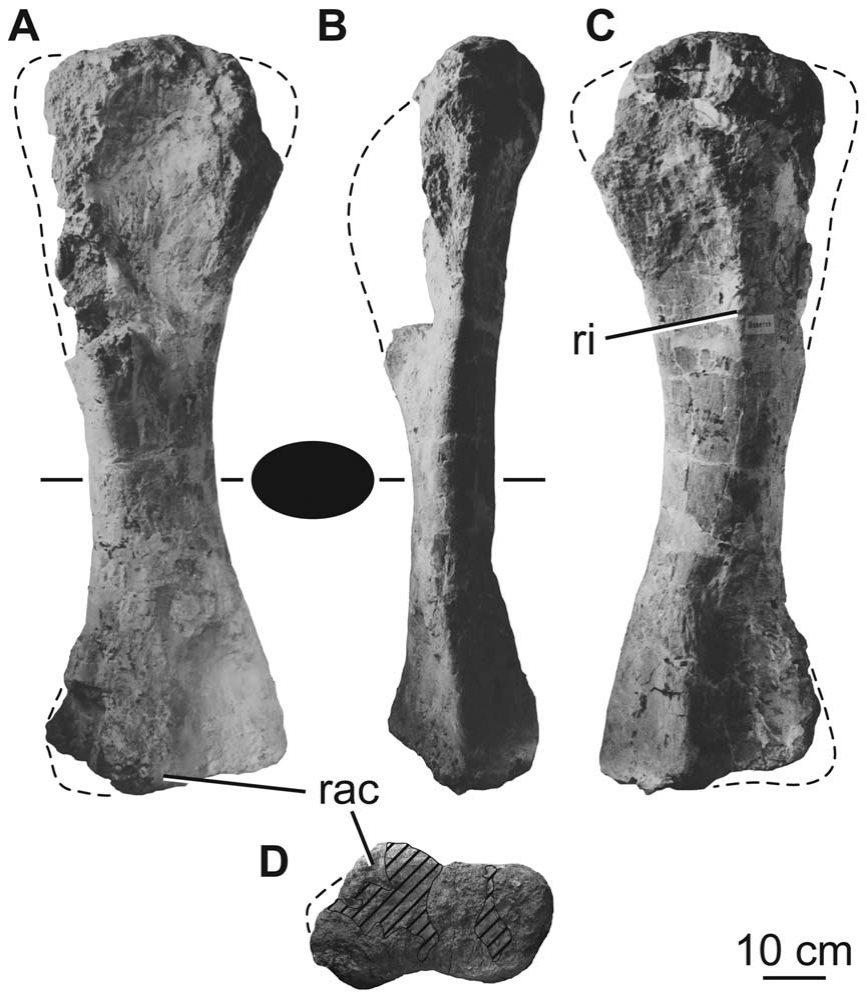
Isolated right humerus (HBV-20002) from the Upper Cretaceous of Shanxi, China. Photographed during original preparation in (A) anterior, (B) medial, (C) posterior, and (D) distal views. This bone was formerly regarded as the paratype of *H. allocotus.* Silhouette between (A) and (B) shows schematic cross section of the diaphysis. *Abbreviations: rac, radial condyle; ri, ridge.* Striped pattern indicates broken surface, dashed lines indicate broken bone margins.

Although the humerus listed as the paratype of *H*. *allocotus* by Pang and Cheng [14] cannot be referred to the species because of a lack of overlapping elements, we provide a brief description here because of its significance (see Discussion below). The humerus is nearly complete, missing only its deltopectoral crest and parts of its proximal and distal ends (Fig. 24). It is gracile (midshaft width divided by length  = 0.16; Table 10), with subequally expanded proximal and distal ends. There is a broad deltopectoral fossa along the proximal half of the anterior surface and a sharp longitudinal ridge posteriorly (Fig. 24). The posterior surface of the distal end forms a deep supracondylar fossa, bounded by sharp ridges, as is the case in many other titanosauriforms [1], [30]. Many phylogenetically informative characters (e.g., squared proximolateral corner; posterolateral bulge on proximal end of humerus) cannot be ascertained in this humerus because of damage.

**Table 10:** Measurements of the left humerus (HBV-20002) from the Upper Cretaceous of China previously referred to as the paratype of *Huabeisaurus allocotus*.

Dimension	Measurement
Length	1220
Proximal end mediolateral width	410
Midshaft minimum circumference	560
Midshaft anteroposterior length	140
Midshaft mediolateral width	200
Distal end anteroposterior width	190
Distal end mediolateral width	390

All measurements are in millimeters.

## Discussion

### Affinities of *Huabeisaurus allocotus*

*Huabeisaurus* is a relatively complete and well-preserved sauropod, with parts of all major regions of the body preserved. The new data concerning *Huabeisaurus* presented above and the wealth of new Cretaceous Asian sauropod discoveries over the past decade prompt a reevaluation of its affinities. A number of named Cretaceous East Asian titanosauriforms await detailed description and comparisons; since we have yet to make first-hand observations of many of these species, our understanding of the phylogenetic relationships of them is limited. We refrain from incorporating *Huabeisaurus* into a cladistic analysis until more detailed data from other taxa are available. However, comments can be made as to its affinities based on synapomorphies recovered in previous cladistic analyses. Below, we evaluate previous hypotheses of affinity between *Huabeisaurus* and other Cretaceous East Asian sauropods.

*Huabeisaurus allocotus* has never been incorporated into a cladistic analysis, but several authors have commented on its affinities on comparative grounds. Buffetaut et al. [4] reviewed the sauropods of Thailand and described Late Jurassic and Early Cretaceous teeth similar to those of euhelopodids (in the traditional sense, i.e., *Euhelopus, Mamenchisaurus* and *Omeisaurus*) and others more reminiscent of *Nemegtosaurus* and *Phuwiangosaurus* (which they termed nemegtosaurids). Following these comparisons, *Huabeisaurus* was regarded as a nemegtosaurid by Buffetaut et al. [4]. Upchurch et al. [30] considered *Huabeisaurus* to represent a titanosauriform or titanosaur outside of Lithostrotia on the basis of a variety of features (see below). You et al. [6] suggested a close relationship between *Huabeisaurus*, *Borealosaurus*, and *Opisthocoelicaudia.* Mo et al. [70] highlighted similarities among *Huabeisaurus*, *Fusuisaurus*, and *Sonidosaurus*. Tanimoto et al. [71] considered *Huabeisaurus* to be closely related to *Nemegtosaurus*, *Borealosaurus* and the “Toba sauropod” of Japan (as members of Nemegtosauridae). Finally, it has recently been suggested [2] that *Huabeisaurus* is a member of Euhelopodidae. Below we reevaluate the evidence for nemegtosaurid, opisthocoelicaudine, and euhelopodid affinities for *Huabeisaurus*.

### *Huabeisaurus* as a nemegtosaurid

Buffetaut et al. [4] suggested that Huabeisaurus is a member of Nemegtosauridae on the basis of similarities shared with Phuwiangosaurus, which they also considered to be a nemegtosaurid. No specific features were mentioned to support nemegtosaurid affinities for Huabeisaurus by Buffetaut et al. ([4]:97), but five were listed to link Phuwiangosaurus and Nemegtosaurus: (1) “narrow premaxillae”, (2) “a broad quadrate fossa”, (3) “a very small supratemporal fenestra”, (4) “a quadrangular frontal”, (5) “and a postorbital with a very long anteroventral ramus”. However, these features are all more widely distributed among sauropods. Because all sauropods have four premaxillary teeth, the breadth of the premaxilla (feature 1) is related to tooth breadth. Nemegtosaurus and Phuwiangosaurus have narrow teeth and therefore narrow premaxillae, but these traits were apparently acquired independently in these taxa (see below) as in other sauropod lineages (e.g., Diplodocoidea versus Titanosauria [12]). Most sauropods have what could be construed as a broad quadrate fossa (feature 2), and its breadth does not particularly link Phuwiangosaurus and Nemegtosaurus [5], [30]. A small supratemporal fenestra (feature 3) characterizes many titanosauriforms [72]. Sauropod frontals are generally quadrangular (feature 4), with four nearly straight sides bordering the orbit and postorbital laterally, prefrontal and nasal anteriorly, parietal posteriorly, and complementary frontal medially, although these borders have varying lengths among sauropods [30]. The length of the anteroventral (jugal) ramus of the postorbital (feature 5) is indeed long in Phuwiangosaurus and Nemegtosaurus (total postorbital length divided by anteroposterior length of proximal body ca. 2.3), but similar values occur in some other sauropods (e.g., Tapuiasaurus [73]).

The dental features cited by Tanimoto et al. [71] in support of nemegtosaurid affinities for Huabeisaurus were refuted by Saegusa and Tomida [74], who pointed out that these features have a wider distribution among sauropods.

Furthermore, nemegtosaurid affinities for Phuwiangosaurus are refuted by several recent cladistic analyses that indicate that Phuwiangosaurus lies outside of Titanosauria [2], [75], [76], with Nemegtosaurus nested within lithostrotian titanosaurs [33], [57], [73]. Therefore, the several similarities shared between Huabeisaurus and Phuwiangosaurus (see “Description” above) are not indicative of nemegtosaurid affinities.

### *Huabeisaurus* as an opisthocoelicaudine

You et al. [6] suggested that *Huabeisaurus*, *Opisthocoelicaudia*, and *Borealosaurus* are closely related within Opisthocoelicaudinae on the basis of opisthocoelous caudal vertebrae. However, this is problematic because the middle caudal vertebrae (ca. position 17–23) of *Opisthocoelicaudia* are not strongly opisthocoelous as in the holotypic middle caudal vertebra of *Borealosaurus*; instead they are amphiplatyan ([77]: pl. 6). *Huabeisaurus* likewise has amphiplatyan caudal vertebrae in this part of the tail and only displays an anterior convexity in two middle-distal caudal centra (Cd28–29; see above). The most common condition for anterior-middle sauropod caudal vertebral centra is one in which the anterior face of the centrum is slightly more concave than the posterior face [78]. Although *Opisthocoelicaudia*, *Huabeisaurus*, and *Borealosaurus* are all unusual with respect to the primitive titanosauriform condition of caudal vertebral articulation, they are unusual in different (i.e., non-homologous) ways; opisthocoely occurs in different parts of the tail and to different degrees in these genera. Several other Cretaceous East Asian genera have caudal vertebral central articular surfaces similar to those of *Huabeisaurus* (i.e., the posterior concavity is deeper than the anterior concavity), including *Tangvayosaurus* (TV2, MDD pers. obs. 2008) and *Phuwiangosaurus* (PW1, MDD pers. obs. 2008; [75]), providing evidence for a close relationship among these taxa.

Another feature recognized herein links elements referred to *Huabeisaurus* and *Borealosaurus*, albeit in a complex manner. The isolated humerus (HBV-2002), formerly the paratype of *Huabeisaurus* (see above), has a longitudinal ridge on the posterior face of the humeral shaft. A comparable ridge also occurs on a humerus referred to *Borealosaurus wimani* ([6]; LPM 0170, MDD, PDM, PU pers. obs. 2012). Unfortunately, like the referral of the humerus HBV-20002 to *Huabeisaurus*, the referral of the humerus LPM 0170 to *Borealosaurus* cannot be substantiated, as the association of the hypodigm of *Borealosaurus* is unknown [6]. Indeed, the humerus referred to *Borealosaurus* seems to be much too small (610 mm long) to belong to the same individual as the holotypic mid-caudal vertebra (112 mm long) based on comparisons with complete sauropods of similar size (e.g., *Rapetosaurus* [37]). Nonetheless, the uniquely shared presence of a posterior longitudinal ridge on these two isolated humeri (HBV-20002, Fig. 24 and LPM 0170) suggests further evidence of close relatedness among Cretaceous East Asian sauropods. Alternately, these two isolated humeri may pertain to the same taxon.

### A new hypothesis: *Huabeisaurus* is a euhelopodid

*Huabeisaurus* appears to be a titanosauriform based on the presence of numerous synapomorphies of the group, including elongate middle cervical vertebrae, anterior and middle caudal vertebral neural arches set on the anterior half of the centrum, middle caudal vertebrae with posteriorly projecting transverse processes, pneumatic and plank-like dorsal ribs, a laterally flared iliac preacetabular process, a pubis at least three times as long as the puboischial contact, and an ischium with a raised tubercle on its posterolateral face [2], [11], [30], [33].

*Huabeisaurus* is a member of Somphospondyli based on the presence of somphospondylous vertebral pneumaticity, anterior dorsal vertebrae with spinodiapophyseal laminae that are low in relief on the front of the neural spine, a scapular glenoid that is beveled medially, and a scapular blade that is straight on the acromial side [2], [12], [30], [33].

A number of anatomical features suggest that *Huabeisaurus* is a member of Euhelopodidae (sensu [2]), a clade that also has been suggested to include *Euhelopus*, *Erketu*, *Daxiatitan*, *Dongyangosaurus*, *Baotianmansaurus*, ‘*Huanghetitan’ ruyangensis*, *Qiaowanlong*, *Tangvayosaurus* and *Phuwiangosaurus* [2]. These features include: bifid cervical neural spines, pendant cervical ribs, enlarged chevron facets on anterior-middle caudal vertebrae, posterior centrum articulation concave with anterior centrum articulation nearly flat in the anterior–middle caudal vertebrae, and an ilium with a low, pointed preacetabular process [2]. Although we provisionally conclude that *Huabeisaurus* is a member of Euhelopodidae, there are some potential caveats that accompany this interpretation. First, placement of *Huabeisaurus* within Euhelopodidae has not yet been supported by inclusion in a formal cladistic analysis. Mannion et al. [79] found support for a monophyletic Euhelopodidae in some of their phylogenetic analyses, however, this clade breaks up to form a paraphyletic grade at the base of Titanosauria when some characters are treated as continuous data. Moreover, *Huabeisaurus* shares some similarities with more derived titanosauriforms (e.g., the distal expansion of the radius). It is therefore conceivable that future phylogenetic analyses will result in separation of the large Euhelopodidae found by D’Emic [2] into several smaller clades distributed among different regions of the somphospondylan evolutionary tree. One of these groups could be Huabeisauridae, with *Huabeisaurus* closely related to forms such as *Tangvayosaurus*, similar to the taxonomic scheme proposed by Pang and Cheng [14]. As noted above, these issues can only be resolved through more extensive first-hand examination of Cretaceous Asian sauropod specimens and augmented phylogenetic analyses.

### Referral of other specimens to *Huabeisaurus*

Pang and Cheng [14] tentatively suggested that ‘*Titanosaurus’ falloti* be referred to *Huabeisaurus*. However,*‘T.’ falloti* is only represented by femoral remains, and its femur bears no uniquely shared features with *Huabeisaurus*. Furthermore, the femora of *Huabeisaurus* and *‘T.’ falloti* differ in the bevel of the distal end versus the long axis of the bone (compare Fig. 21 with fig. 17 in [80]), so the two cannot represent the same genus.

### The autapomorphic tibia: femur ratio of Huabeisaurus

**Figure 25:**
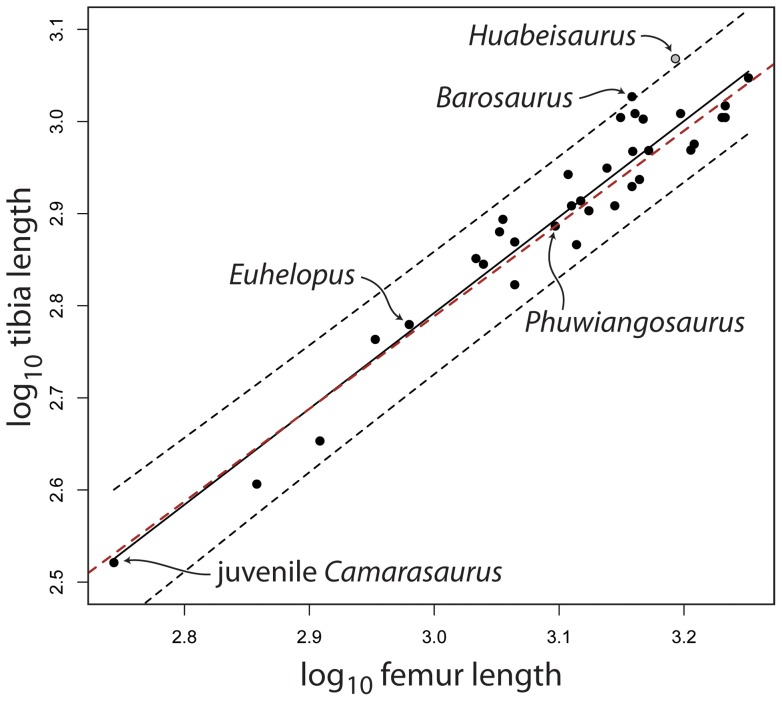
Tibia to femur ratio in sauropods. Linear regression (solid black line) and 95% confidence intervals (dotted lines) are shown, as well as pGLS line (red dashed line). Note that *Huabeisaurus* has an unusually high tibia to femur ratio, only approached by some distantly related diplodocines.

The ratio of the length of the tibia to that of the femur is approximately 0.75 in *Huabeisaurus*. We tested whether this was autapomorphically high compared to other sauropods given possible allometric scaling relationships, initially by performing ordinary least squares regression of log_10_ (tibia length) against log_10_ (femur length) for a sample of 34 other sauropod individuals (Fig. 25; see File S1 for data) using R version 2.15.1 [81] (see File S2 for R code). The regression equation is:
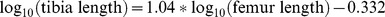



Confidence intervals (95%) about the coefficient (slope) of log_10_ (femur length) are 0.94 to 1.14, so isometry of sauropod hindlimb proportions cannot be rejected. Confidence intervals about the y-intercept are −0.644 to −0.019. Using the above equation, the femoral length of *Huabeisaurus* predicted a log_10_ (tibia length) of 2.99 log_10_(mm), with 95% confidence intervals of 2.93–3.06 (Fig. 25) that do not contain the actual value of 3.07 log_10_(mm).

We performed a second ordinary least squares analysis in which individuals of *Apatosaurus* (N = 2), *Camarasaurus* (N = 6), *Omeisaurus* (N = 3), and diplodocines (N = 6) were collapsed to mean values. The regression equation for this reduced sample size (N = 21) is:
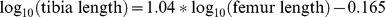



Confidence intervals are 0.855 to 1.11 for the coefficient and −0.568 to 0.238 for the y-intercept. *Huabeisaurus* has a predicted log_10_ (tibia length) of 2.98 log_10_(mm), with 95% confidence intervals of 2.92–3.03, also excluding the actual value of 3.07 log_10_(mm).

Some diplodocines from the Late Jurassic Morrison Formation approach the tibia: femur ratio observed in *Huabeisaurus* (Fig. 25). However, because diplodocines are only distantly related to *Huabeisaurus*, we interpret the ratio in *Huabeisaurus* as an autapomorphy.

Finally, to model the expected covariances among specimens as part of our regression analysis, and thus circumvent problems of phylogenetic relatedness of the data, we performed phylogenetic generalized least squares regression (pGLS; [82]) based on our specimen-level data (N = 34) using a composite phylogeny based on previous cladistic studies ([2], [33]; see Files S3 and S4 for phylogenies at the generic and specific levels, respectively). Branch lengths were calibrated using geologic time (with a minimum length of 0.01 Ma among specimens of *Apatosaurus*, *Camarasaurus*, *Omeisaurus*, and *Diplodocus* and *Barosaurus*). Lambda, a measure of phylogenetic signal [83], was estimated as part of the model fitting process using the R packages nlme version 3.1–104 [84] and ape version 3.0–5 [85]. The maximum likelihood estimate of lambda (0.68) indicates an intermediate level of phylogenetic signal in the relationship between log_10_− femoral length and tibia length (when lambda  = 1.0 branch lengths are left unaltered; when lambda  = 0 scales the tree to a star phylogeny, representing zero phylogenetic signal). Estimates of the coefficient and y-intercept of the pGLS regression line were within their 95% confidence intervals derived from ordinary least squares regression (coefficient  = 1.01, y-intercept  = −0.230). Importantly for our purposes, the phylogenetically informed regression line passes further from *Huabeisaurus*, confirming that its hindlimb proportions are indeed autapomorphic (Fig. 25).

### Tooth shape in Euhelopodidae

**Figure 26:**
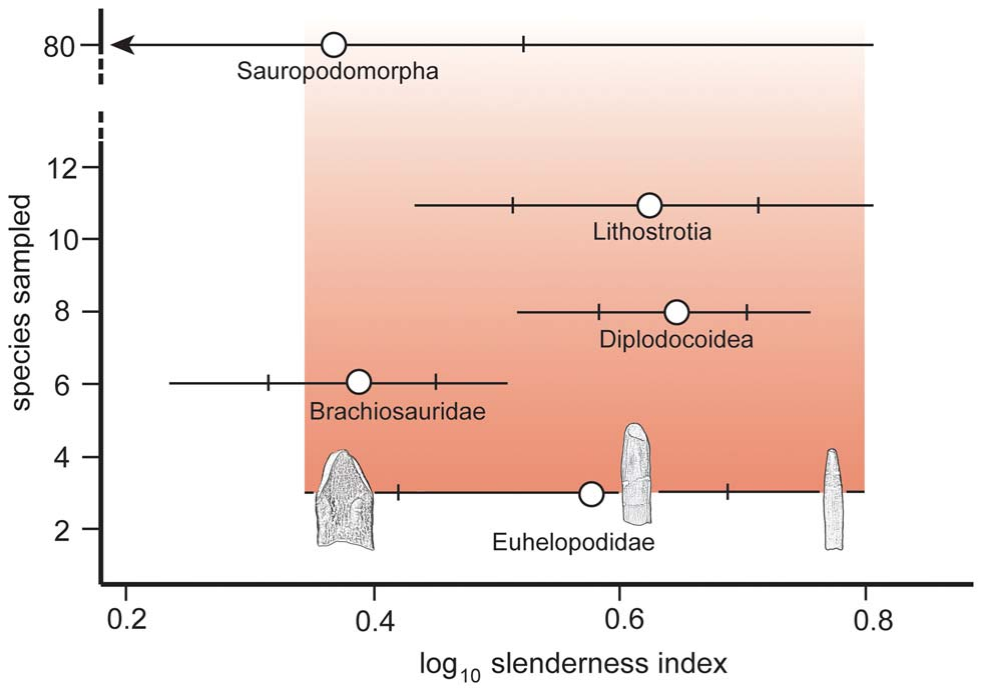
Crown slenderness index versus number of genera known from teeth in Sauropodomorpha and derived clades. The mean (white circle), standard deviation (tick mark) and range (horizontal line) of tooth breadth for each clade is indicated (log_10_ Sauropodomorpha slenderness index range extends towards zero as indicated by the arrow). Sketches of tooth crowns (from left to right, *Euhelopus, Huabeisaurus, Phuwiangosaurus*) and red field depict the wide range of crown shapes in Euhelopodidae compared to other derived sauropod clades.

Euhelopodids (sensu [2]; e.g., *Euhelopus, Phuwiangosaurus, Tangvayosaurus*, *Erketu*) are united by axial and appendicular synapomorphies and have a notable diversity of tooth shapes. Figure 26 plots the slenderness index (SI; crown length divided by maximum crown breadth) for four derived sauropod clades (Diplodocoidea, Brachiosauridae, Euhelopodidae, and Lithostrotia; data modified from [29]; see File S5 for data) versus the number of genera that have teeth included in the hypodigm. Euhelopodidae (inclusive of *Huabeisaurus*) shows the widest range of tooth breadth of any of these clades, despite having the smallest sample. Within Euhelopodidae, the teeth of *Euhelopus* have low slenderness indices (mean SI of all teeth  = 2.50), close to the mean for Sauropodomorpha (2.32). In contrast, *Phuwiangosaurus* has very slender teeth (mean SI of all teeth  = 4.37), which is close to the mean for Diplodocoidea (4.46). *Huabeisaurus* teeth are intermediate in SI (mean SI  = 3.41). Although relationships within Euhelopodidae are uncertain, it is clear that narrow tooth crown breadth evolved at least once in the clade. This represents a fourth example of the evolution of narrow crowns in sauropods, along with diplodocoids, brachiosaurids, and titanosaurs [29]. Tooth breadth varies with inferred feeding strategy and perhaps diet in sauropods [29], [86]. We preliminarily interpret the wide range of tooth breadths in Euhelopodidae as evidence for a broad range of feeding strategies and/or diets within the clade.

## Conclusions

Redescription of *Huabeisaurus allocotus* from the Upper Cretaceous of Shanxi, China confirms its validity and membership within the Somphospondyli. *H. allocotus* is diagnosed by a set of unique features in the axial and appendicular skeleton. *Huabeisaurus* is not a member of Nemegtosauridae or Opisthocoelicaudinae as previously suggested. Instead, a number of features are shared between *Huabeisaurus* and *Euhelopus, Phuwiangosaurus*, and *Tangvayosaurus*, suggesting that *Huabeisaurus* is a late-surviving member of Euhelopodidae. Only a few euhelopodids are known from dental remains, but these teeth exhibit an exceptional range of morphologies relative to other sauropodomorphs, suggesting broad ecological diversity among Euhelopodidae as a whole.

## Materials and Methods

Specimens were observed first-hand by all authors at the Geological Museum of Shijiazhuang University in March 2012. Measurements were made to the nearest millimeter with digital calipers and measuring tapes. Permission to view the collections of the Geological Museum of Shijiazhuang University was obtained from D. Hao. Because some of the bones are now extensively covered with plaster, we supplemented our study with archival photos taken of each bone during original preparation. These photos are housed at the Geological Museum of Shijiazhuang University. Specimens were collected by Pang and Cheng, and were not purchased, donated, or loaned.

## Supporting Information

File S1 Datafile with sauropod tibia and femur lengths (in millimeters). (TXT)

File S2 R code for performing tibia: femur regressions. (TXT)

File S3 Phylogeny of sauropods at the genus level with branch lengths scaled against geologic time. (PHY)

File S4 Phylogeny of sauropods at the species level with branch lengths scaled against geologic time. (PHY)

File S5 Slenderness indices (crown length divided by maximum crown mesiodistal breadth) for sauropod teeth. (XLS)
